# Bayesian analysis of Ecological Momentary Assessment (EMA) data collected in adults before and after hearing rehabilitation

**DOI:** 10.3389/fdgth.2023.1100705

**Published:** 2023-02-17

**Authors:** Arne Leijon, Petra von Gablenz, Inga Holube, Jalil Taghia, Karolina Smeds

**Affiliations:** ^1^ KTH - Royal Institute of Technology, Stockholm, Sweden; ^2^ Institute of Hearing Technology and Audiology, Jade University of Applied Sciences, Oldenburg, Germany; ^3^ ORCA Europe, WS Audiology, Stockholm, Sweden

**Keywords:** Ecological Momentary Assessment, EMA, ambulatory assessment, experience sampling, Bayesian inference, ordinal data, nominal data

## Abstract

This paper presents a new Bayesian method for analyzing Ecological Momentary Assessment (EMA) data and applies this method in a re-analysis of data from a previous EMA study. The analysis method has been implemented as a freely available Python package *EmaCalc*, RRID:SCR 022943. The analysis model can use EMA input data including nominal categories in one or more situation dimensions, and ordinal ratings of several perceptual attributes. The analysis uses a variant of ordinal regression to estimate the statistical relation between these variables. The Bayesian method has no requirements related to the number of participants or the number of assessments by each participant. Instead, the method automatically includes measures of the statistical credibility of all analysis results, for the given amount of data. For the previously collected EMA data, the analysis results demonstrate how the new tool can handle heavily skewed, scarce, and clustered data that were collected on ordinal scales, and present results on interval scales. The new method revealed results for the population mean that were similar to those obtained in the previous analysis by an advanced regression model. The Bayesian approach automatically estimated the inter-individual variability in the population, based on the study sample, and could show some statistically credible intervention results also for an unseen random individual in the population. Such results may be interesting, for example, if the EMA methodology is used by a hearing-aid manufacturer in a study to predict the success of a new signal-processing method among future potential customers.

## 1. Introduction

Driven by the rapid development in smartphone technologies, Ecological Momentary Assessment (EMA), also known as Ambulatory Assessment or Experience Sampling, has become a popular method particularly in behavioral research. The main motivation is that EMA is expected to achieve better ecological validity than conventional tests performed in a laboratory (1). In EMA, each study participant is requested to respond to an electronic questionnaire during everyday life, typically several times per day. Some questions may address the current *real-life situation*, for example, the physical environment and the user’s activity and intentions in that particular situation. The participant may also be asked to rate various *perceptual attributes* of interest in the study. Most conveniently, the questions are presented and responses collected by a special-purpose app installed on a smartphone. The timing of assessments may be determined randomly by the app, and users may also be allowed to initiate an assessment at any time of their own choice.

Although EMA is a general method that can be used in many scientific fields, we now focus on applications in audiology. In this context, EMA is often used to investigate the benefit and subjective quality of hearing instruments in the user’s real life (e.g., (2–7)). So imagine the purpose of an EMA-based study is to evaluate, for example, a new hearing aid in comparison to a base-line reference. Then the goal of a statistical analysis is to determine whether the intervention yields a true benefit, such that an apparent improvement is not just a random effect caused by a limited number of study participants or a limited number of assessments by each respondent. In other words, the researcher needs to determine the statistical credibility of any observed changes in EMA response patterns. Here, the word “credibility” can mean three quite different quantities:

1. The predictive probability that the result is true for an unseen *random individual* in the population from which the study participants were recruited.
2. The predictive probability that the result is true for the *mean (or median) in the population* from which the study participants were recruited.
3. The probability that the result is true for each and any *participant* in the study.

The first of these probability measures may be most important in, for example, a study designed by a hearing-aid manufacturer to predict the success of a new product among future customers. The second probability measure is most closely related to the statistical significance as estimated by conventional hypothesis tests. The third measure might be most important in a clinical study to quantify the benefit for individual clients.

Against this background, the present report proposes a Bayesian probabilistic model and presents a freely available python package *EmaCalc*, RRID:SCR_022943, that can help researchers meet the challenges of EMA data analysis. This work is not a comparative study of Bayesian versus frequentist approaches. We do not claim that *EmaCalc* is generally superior to existing advanced frequentist analysis methods for ordinal and nominal data, although we think the Bayesian approach offers some advantages, and the package is easy to use. We applied this new tool in a re-analysis of data from the study “Individual Hearing Aid Benefit in Real-Life (IHAB-RL)” (6), which included heavily skewed, scarce, and clustered ratings that were collected on ordinal scales. The results will show effects of a hearing-aid fitting intervention on subjective judgements of *Loudness*, *Involvement* in group conversations, and *Disability*.

## 2. Challenges of EMA data analysis

The data set recorded in an EMA study presents several statistical challenges, most of which were discussed in depth by Oleson et al. (8):

• There may be a large amount of data from each participant, but the number of assessments can vary greatly among respondents.
• Some of the responses are nominal (categorical). For example, the current real-life situation may be characterized in several dimensions, e.g., as “indoors”/“outdoors” and “quiet”/“noisy,” etc. The respondent might also report which hearing-aid program was active.
• Other responses are ordinal. For example, the respondent may rate the “Disability” by selecting one response from a range of discrete alternatives like “not disabled at all,” “very slightly,” “slightly,” “moderately,” “considerably,” “very” or “extremely” disabled (6). The number of ordinal response alternatives may differ among questions.
• With many assessments by each participant, the responses collected in total from all participants are not statistically independent. Therefore, a multi-level approach is needed, such that the responses are analyzed as nested within each individual, separate from the next level of variability across respondents.
• Typically, the ordinal ratings can not be encoded numerically to represent points on an interval scale: We cannot take it for granted that the steps between response categories are perceptually equal in magnitude. Therefore, it may be questionable to aggregate responses within individuals by conventional measures such as mean and variance of integer-encoded ordinal ratings.
• Individual respondents might interpret and use the ordinal response scale in different ways. For example, some people might tend to use the more extreme response alternatives, while others hesitate to do so (9). Therefore, it may be questionable to assume that ordinal responses have the same quantitative meaning for everyone in a population.
• The subjective benefit of an intervention, such as hearing instrument provision, may change with the real-life environment. Therefore, it may be interesting to analyze the ordinal responses about perceptual attributes as conditional on the nominal responses to the situation questions.
• The responses about the real-life situation might also show the benefit of an intervention. For example, if response patterns indicate that hearing-aid users are more likely to visit a challenging sound environment when using a particular hearing-aid program, this might be interpreted as a benefit of the signal processing methods in that program.
• A complete study may be designed to include two or more test phases, yielding separate series of EMA data. For example, participants may be asked to record baseline responses first without hearing aids, and then to record a similar follow-up series a few months later, after adaptation to their new hearing aids (e.g., (6)).

It has been overwhelmingly common in the behavioral-science literature to apply conventional statistical measures and models such as mean and variance, t-test, ANOVA, linear or non-linear regression, using the raw subjective ratings as input, although all these analysis methods are *metric*, i.e., they presume that the input data have interval-scale properties. The main reason is that these conventional methods are readily available in statistical program packages, so most researchers are experienced in using them and interpreting their results. Considering the trade-off between methodological appropriateness and interpretatbility, Oleson et al. (8) suggested that general-purpose statistical approaches such as linear or generalized-linear mixed regression models (GLM) can sometimes adequately capture the essential characteristics of EMA data, in spite of the complex nature of the data. However, they also noted that analyzing ordinal data as if they were metric can cause errors. The errors might even have severe scientific consequences, such as indicating a statistically significant effect in the wrong direction (10). These potential errors cannot be detected by calculations such as conventional normality tests. The only way to detect an error would be to compare the analysis results with those of another model that does not presume metric data. The GLM framework can be extended to handle ordinal data, for example, as in the frequentist Cumulative Linear Mixed Model (CLMM) used by von Gablenz et al. (6) for the IHAB-RL data. Oleson et al. (8) also suggested that the full complexity of EMA data might be handled properly by a hierarchical Bayesian model, but noted that there is currently no software readily available that can perform this type of analysis.

We now propose the Python software package *EmaCalc*, RRID:SCR_022943 (https://www.pypi.org/EmaCalc) as a tool to fill this gap. The following Sections 3.1 and 3.2 will present an overview of the theoretical model, Section 3.3 discusses the usage of the software, while Sections 3.4 and 4 present data and results from the IHAB-RL study. All mathematical details of the model, the Bayesian learning method, and the implementation, are specified in a Supplement “EmaCalc Math Details” to this paper.

## 3. Method

### 3.1. Bayesian versus frequentist

Although researchers using EMA might be most familiar with the conventional frequentist viewpoint of statistics, Bayesian methods have recently gained increased adoption in the social sciences and require a slightly different way of thinking (11). The main advantage of the Bayesian approach is that the statistical analysis *“will usually not result in a single estimate, but will yield a range of estimates with varying plausibilities associated with them”* (11).

For example, in the frequentist framework we can calculate a 95% confidence interval for some unknown attribute value, based on a set of observed data. However, this does not mean that there is 95% probability for the true attribute value to fall within the confidence interval. In the frequentist view, it is only the observed data that are assumed to be drawn at random from some probability distribution family, but the true value is just a fixed unknown number without any associated probability distribution. Therefore, the confidence interval is a random outcome, because its end points are deterministic functions of the observed data, but the true value is still just an unknown number. The confidence interval is useful for practical purposes, because we know it has been calculated by a method that is expected to include the true value in about 95% of all similar studies where the same analysis method is used.

In contrast, the Bayesian approach estimates an explicit probability distribution for the true attribute value, given the observed data set. The resulting probability distribution can be described in many ways. For example, we can present the 2.5- and 97.5-percentiles of the obtained cumulative probability distribution for the true attribute value. The Bayesian *credible interval* between these percentile points by definition contains 95% of the probability mass for the true attribute value. In this sense, the credible interval can be used for practical purposes in a similar way as the frequentist confidence interval, but the Bayesian approach has a different theoretical background.

There is a similar theoretical issue in statistical hypothesis testing. Assume a study was designed to test a hypothesis that A<B as opposed to A≥B, where A and B can be effects of two different interventions, or some perceptual attribute value in two test conditions. In a frequentist data analysis, we must start from the null hypothesis, A=B, and then show that there is a very small probability (e.g., less than 5%) to obtain the observed result, or a more extreme result, if the null hypothesis were true. However, we still do not know the probability P(A<B), because no probability measure is defined for the hypotheses themselves.

In contrast, the Bayesian approach yields explicit numerical probability values P(A<B) and P(A≥B), given all observed data. Once the Bayesian analysis has derived a joint probability distribution for all model parameters, it is also easy to calculate the joint probability for multiple hypotheses combined, with no need for further adjustments like the classical Bonferroni correction. Interesting philosophical and historical discussions of the Bayesian and frequentist perspectives are given in the literature (e.g., (11–13)).

### 3.2. EmaCalc: theory

#### 3.2.1. Notation and model overview

For the EMA data analysis, let us now assume a study that involves N participants,^1^ all describing the situation at each assessment by a choice among K nominal categories. The participants may also give an ordinal rating for each of I questionnaire items about perceptual attributes evaluated in the study. The study may include T≥1 test phases, but the situation categories and attribute items are the same for each test phase. Assume we have collected $R_{n}$ complete EMA reports from the nth participant, with each report identified by an index $r\in \{1,\ldots ,R_{n}\}$.

The mathematical derivation presented in Supplementary Section A shows that the log-likelihood of all recorded EMA data from the nth participant can be calculated as a function of an individual parameter vector $\xi_{n}=(\xi_{n1},\ldots ,\xi_{nD})$. This parameter vector includes three separate classes of model parameters:

1. Parameters specifying the *situation probabilities*, i.e., the probability for the respondent to visit different situations.
2. Regression parameters specifying the *situation effects*, i.e., how the attribute ratings tend to differ across situations.
3. Parameters defining the ordinal *response scale* for each attribute question.

The distribution of individual parameter vectors in the population, from which participants were recruited, is specified by a separate population model. The number of EMA recordings may vary among participants. If one participant provides unusually many EMA records, this will tend to improve the precision of the individual parameter estimate for that person, but this participant will still automatically have the same weight as every other participant in the estimation of population characteristics.

#### 3.2.2. Nominal EMA situation responses

The model assumes that the occurrence of various situations in the EMA records are determined by fixed but unknown *situation probability* vectors $u_{nt}=(u_{nt1},\ldots ,u_{ntK})$. Here, $u_{ntk}\in [0,1]$ is the probability that the nth participant reports from the kth situation category at any assessment in the tth test phase. Situations may be defined as a combination of categories from one or several situation dimensions, as mentioned in Section 2. For example, if one dimension is “location,” with two alternatives, “indoors, outdoors,” and another dimension is “noisiness” with three categories “quiet, moderate, loud,” the total number of situation categories would be K=2×3=6.

The array of situation probabilities is one of the main results to be estimated from the data. The individual situation probabilities are assumed to be the same for all EMA records from the same test phase, but may vary between participants and between test phases.

#### 3.2.3. Ordinal EMA attribute ratings

The ordinal rating responses are analyzed with a variant of Item Response Theory (IRT). IRT is a family of probabilistic models designed to handle issues common to test instruments for any purpose in social, psychological, or educational research. The variant implemented in *EmaCalc* includes individual parameters that can account for the possibility that different people use the ordinal response scales in different ways.

There is a rich literature on IRT, including several text books (e.g., (14,15)) with good reviews of the literature. The *Graded Response* IRT model (16), also called *Cumulative Model* (17,18), is mathematically very closely related to signal-detection theory and choice models that have a long history of use in psycho-acoustical research (e.g., (19–22)). These models are sometimes called “ordinal-probit” or “ordinal-logit.”

The basic feature of ordinal rating models is that subjective responses are regarded as indicators (“symptoms”) that are only probabilistically related to the individual attribute value that is to be measured. The true individual attribute cannot be directly observed. It can only be estimated on the basis of test responses. The model treats each response as determined by an outcome of a latent random variable. The location (mean or median) of the probability distribution of that latent variable is the individual attribute characteristic to be estimated, whereas the response probabilities also depend on other parameters that may differ among respondents.

**Figure 1:**
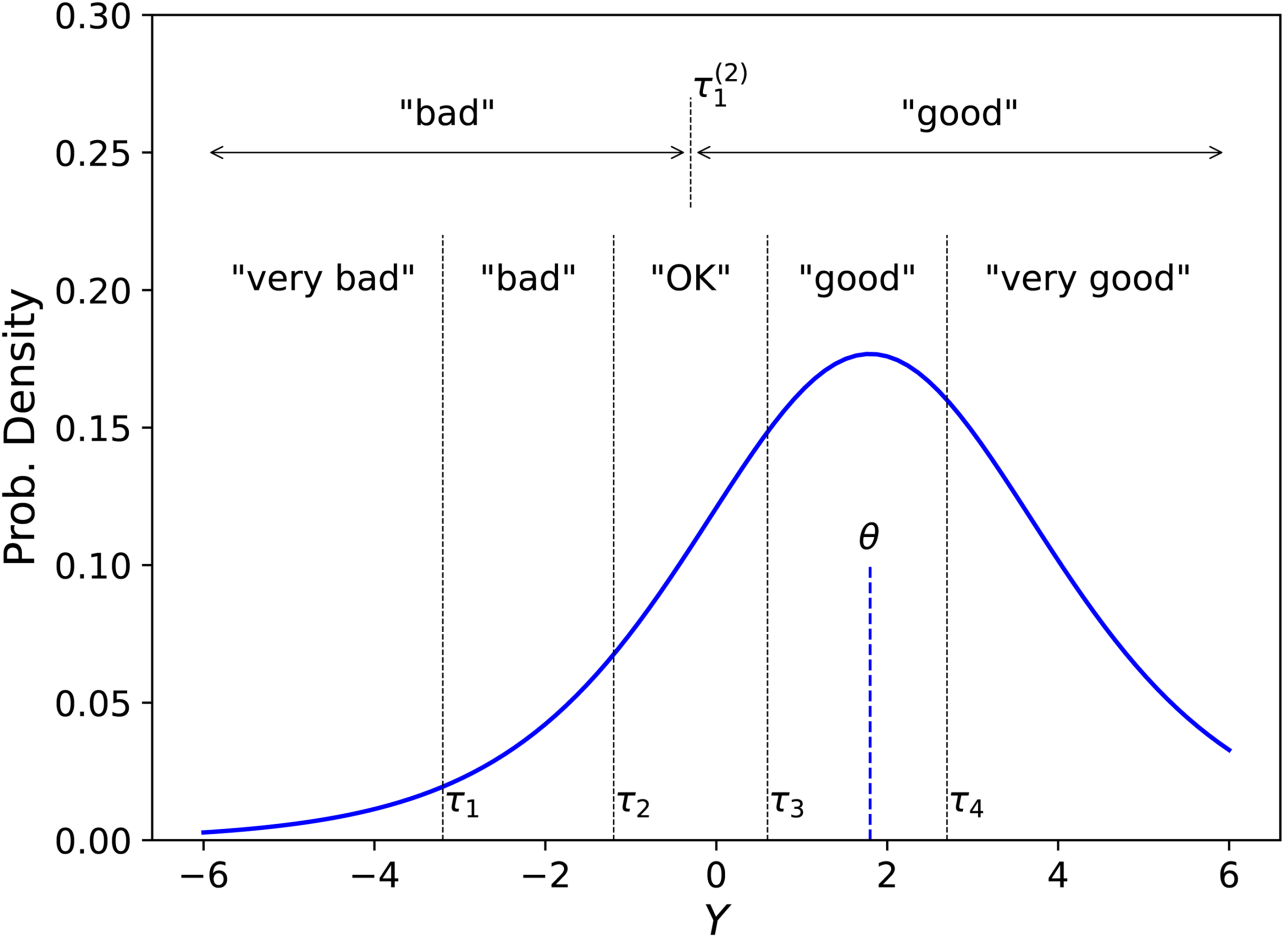
Example of a conditional probability density function of the latent random variable Y whose outcomes determine responses to a question about a perceptual attribute, e.g., Speech Understanding, given a true location parameter θ=1.8 on the logit scale. Response intervals are exemplified for one study allowing five ordinal response categories, “very bad,” “bad,” …, “very good,” with response thresholds $\left(\tau_{1},\tau_{2},\tau_{3},\tau_{4})=(-\;3.2,-\;1.2,0.6,2.7\right)$, and for a second study evaluating the same attribute with only two allowed responses, “bad” or “good,” with a single response threshold $\tau_{1}^{\left(2\right)}=-0.3$. The present model allows separate location parameters θ for each participant, attribute, and situation. The model allows different response thresholds for each participant and attribute, but the thresholds are identical across situations.

In the model, illustrated in Figure 1, each ordinal response is determined by an outcome of a continuous real-valued latent random variable $Y_{nik(r)}$, with a probability distribution specific for the ith attribute of the nth participant in the kth situation (as reported in the rth EMA record). The lth ordinal response is given whenever the latent variable falls in an interval $\tau_{ni,l-1}<$ $Y_{nik(r)}\leq \tau_{ni,l}$, where the thresholds separating the intervals form an increasing sequence $\left(-\infty =\tau_{ni,0}<\tau_{ni,1},\ldots ,<\tau_{ni,L_{i}}=+\infty \right)$. The thresholds may differ between respondents^2^ and between attribute items,^3^ but are assumed to be identical in all assessments of the same attribute by each respondent in all situations. The latent variable is drawn from a logistic^4^ probability distribution with location $\theta_{nik}$ and unity^5^ scale. The location $\theta_{nik}$ is the desired outcome measure for the perceptual attribute that is to be estimated from the observed data. Although all responses are only discrete and ordinal, the attribute value $\theta_{nik}$ is continuous on an interval scale. Thus, the unit of the resulting interval scale is defined by the fixed scale parameter of the latent-variable distribution. This part of the EMA model is very similar to a previous model for paired-comparison data (23) with the Bradley-Terry-Luce (BTL) “ordinal-logit” choice model^6^ (19), i.e., assuming a logistic distribution for the latent sensory variables.

An ordinal regression model is used to estimate how the attribute values tend to differ across situations. These *situation effects* may be specified either (1) to include only a linear combination of *main effects* of the test phase and categories in each situation dimension, or (2) to also include *interaction effects* between the test phase and any combination of categories in separate situation dimensions.

Since this regression model allows both the perceptual attribute value $\theta_{nik}$ and the threshold parameters $\tau_{ni,l}$ to be freely variable for each respondent, the model is under-determined: If a fixed constant value is added to all $\theta_{nik}$ and all $\tau_{ni,l}$, the probability of observed responses does not change. Thus, the zero point on the attribute scale is arbitrary. To make the model identifiable, the zero point must be somehow restricted. The current implementation allows the researcher either (1, default) to force the median response threshold to zero, or (2) to force the average attribute value to zero, for each respondent and each attribute.

#### 3.2.4. Individual and population models

The nominal and ordinal response patterns are assumed probabilistically determined by the individual parameter vectors $\xi_{n}$. Of course, these parameters cannot be directly observed. Their values must be estimated from the recorded data. In the Bayesian framework, all these parameters are regarded as random variables.

**Figure 2:**
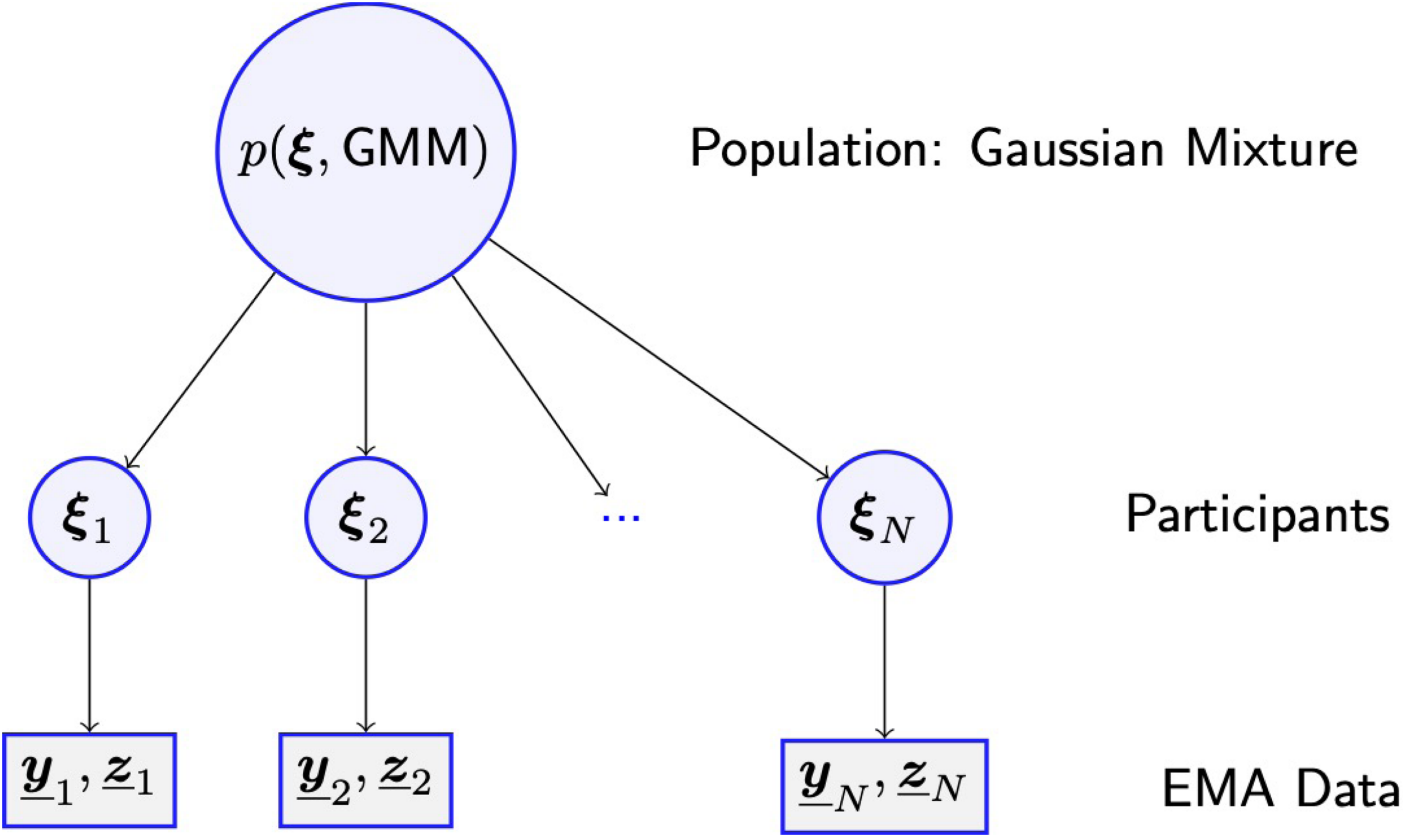
A hierarchical model is assumed to determine probabilities of all observed EMA data, including nominal responses ***z***_*n*_ about the listening situation and ordinal responses ***y***_*n*_ about perceptual attributes in the current situation, for the nth participant. The response probabilities are calculated as functions of a parameter vector $\xi_{n}$ which is regarded as a sample from the distribution of parameter vectors in the population from which the participants were recruited. Using all observed data, the Bayesian learning algorithm estimates probability distributions for parameters $\xi_{n}$ of each participant and the distributions of parameters ξ in the population, as well as for parameters specifying the population Gaussian Mixture Model (GMM).

However, as all participants were recruited at random from the same population, as defined by the researcher, the model treats each individual parameter vector $\xi_{n}$ as a sample drawn at random from a population distribution specified by another set of parameters, as defined in Supplementary Section B. In this way the population model acts as a prior distribution for all individual parameters. The structure of this hierarchical model is illustrated in Figure 2.

The distribution of individual parameter vectors in the population might have been formulated as, e.g., a multivariate Gaussian (normal) distribution with full covariance matrix, i.e., with a very large number of parameters to be estimated. For the present purpose it is proposed instead to use a Gaussian Mixture Model (GMM). Mixture models have the advantage that they can represent arbitrarily complex non-linear dependencies between vector elements, not only ordinary correlations,^7^ even if each mixture component only allows independent (uncorrelated) elements. Another advantage is that the model complexity is automatically determined by the general *Occam’s Razor* feature of Bayesian learning: After learning, the mixture model might automatically come out as including only a single Gaussian component with independent elements (if this is sufficient for the given data set) or as a multi-component mixture (if the data indicate a more complex statistical dependency among the parameters).

If the respondents were recruited from separate populations, e.g., younger and older people, a complete separate GMM is learned for each population, using the data from the corresponding group of participants.

#### 3.2.5. Variational model inference

As described in the Supplement Section C, variational learning (e.g., (24, Ch. 10)) is used to derive approximate posterior probability distributions for the parameters of each participant, as well as a separate posterior distribution for the parameters of the population GMM, given all observed data.

The individual distributions are estimated using the response data from each participant, but these individual estimates are also somewhat regularized by the population model: If the response pattern from one respondent deviates a lot from the patterns of many other participants, the hierarchical model will tend to “explain” any such extreme deviations as a random effect of the limited number of responses rather than as an extreme deviation in the true individual characteristics.

This regularization also handles cases of missing data. It is not too rare that an individual might produce very few responses (or even no response at all) in some situations, especially if the study defines many situation categories. In such a case, the distribution of individual parameters will be influenced mostly by the population model. The population model will still be determined by all data that is available from all respondents.

The population model and all individual models are jointly adapted to all response data together with a weakly informative prior density defined in the Supplementary Section B.2 for the population parameters. The combined hierarchical model automatically includes measures of the uncertainty of each individual result as well as the inter-individual variability in the population.

#### 3.2.6. Predictive distributions

The individual and population models can be used to calculate three desired model-parameter distributions as defined in the Supplementary Section D,

1. for an unseen *random individual* in the population from which a group of participants was recruited,
2. for the *population mean*,
3. for each study *participant*.

These distributions are also used to evaluate the *joint probability* for any combination of hypotheses about differences between attribute values and/or situation probabilities. This calculation, as described in (25, Appendix C), accounts for the effect of joint comparisons, so no further corrections for multiple hypothesis tests are needed.

### 3.3. EmaCalc: usage

*EmaCalc* can be used by anyone with a basic knowledge of the Python language. A supplied template script runs a complete analysis which always includes the following steps:

1. *Setup* an experimental framework, specifying
  • study phases, if more than one,
  • situation dimensions, with nominal categories in each dimension,
  • attributes, with ordinal response categories for each attribute
2. *Load* EMA data, by specifying
  • a directory containing all recorded data files,
  • input file format(s),^8^
  • participant groups, if more than one.
3. *Learn* the probabilistic model, after specifying
  • effects to be included in the ordinal regression model,
  • a time limit for the computation.
4. *Display* results, selecting
  • situation probabilities to be shown,
  • attribute values to be shown versus phase and/or situation categories,
  • results for random individual and/or population mean and/or individual participants,
  • optional properties for result figures and tables.
5. *Save* all results, selecting
  • a directory for all result files
  • figure file format(s) and table file format(s).^9^
  The code package *EmaCalc* RRID:SCR_022943, is freely available on PyPi: https://pypi.org. It uses a custom-made sub-package for Hamiltonian sampling, also freely available with open source on PyPi. The package and its required sub-packages can be installed in the user's Python environment by the command
  python3 -m pip install --upgrade EmaCalc
  The present results were produced by version 0.9.3 of *EmaCalc*.
  To reproduce the analysis results presented in this paper, the reader should contact the second author to access the data base, and then run the script run_IHAB.py which can be obtained by request to the first author.
  This code script is adapted from the general template script run_ema.py included in *EmaCalc*. The package also includes simulation functions and a script run_sim.py, which allows the user to quickly produce a simulated data set, save the data in files as if produced in a real experiment, and run the analysis on the simulated EMA data set.

### 3.4. EMA data

We have used the *EmaCalc* package to re-analyze data collected in the study “Individual Hearing Aid Benefit in Real-Life (IHAB-RL)” (6). A total of 24 adults with mild-to-moderate hearing loss collected EMA data using the open-source EMA system olMEGA (26).

The present re-analysis includes data only for 13 first-time hearing-aid users who collected EMA data without hearing aids as well as after a period of HA adaptation, i.e., in two study phases, with a duration of four days each. The participants described everyday listening situations complemented by subjective ratings of different hearing-related attributes. The description of listening situations allowed a retrospective classification as one of seven CoSS^10^ intention and task categories (27). A total of 1330 EMA records were collected from these 13 participants. Further details are presented in the original publication (6).

**Figure 3:**
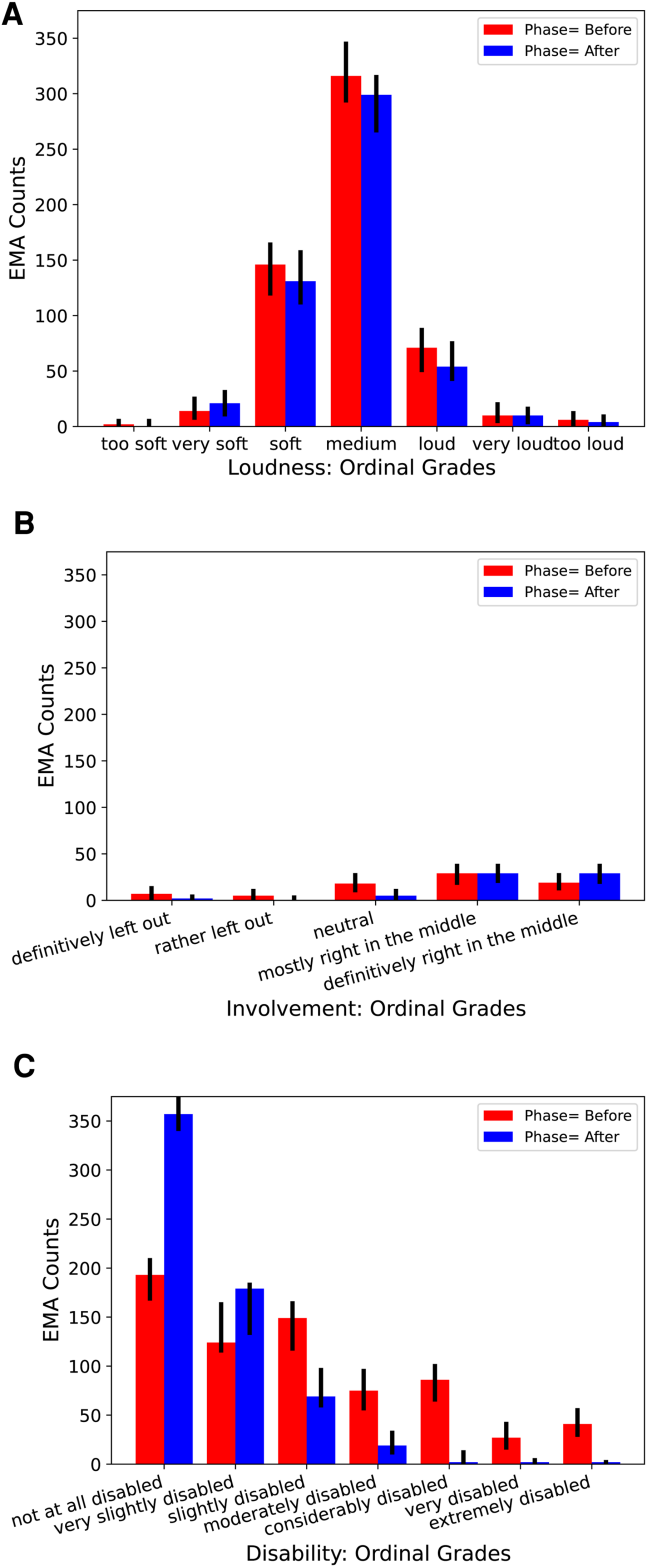
Histograms of ordinal ratings of (**A**) Loudness, (**B**) Involvement in group conversations, and (**C**) Disability in the two phases of the IHAB-RL study, summed across all CoSS categories and all 13 participants in two study phases, before and after hearing-aid fitting. Short vertical black lines on top of the bars show corresponding model-predicted 95% credible intervals for the response counts.

The re-analysis will exemplify how the *EmaCalc* approach can estimate effects of a hearing-aid fitting intervention at the three separate levels mentioned in the Introduction and in Section 3.2.6. Three of the rated attributes are now considered: *Loudness*, *Involvement* in group conversations, and *Disability*. Figure 3 shows histograms of the raw ratings for these attributes. These attributes were selected because the distributional properties of the corresponding data differ greatly from each other: *Loudness* ratings show a comparatively low dispersion, *Involvement* has a scarce data basis with only 143 valid records, and the *Disability* ratings are strongly skewed. As a check to verify that the learned model fits the raw data, the corresponding model-predicted response counts are also shown in the histogram plots.

## 4. Results

**Figure 4:**
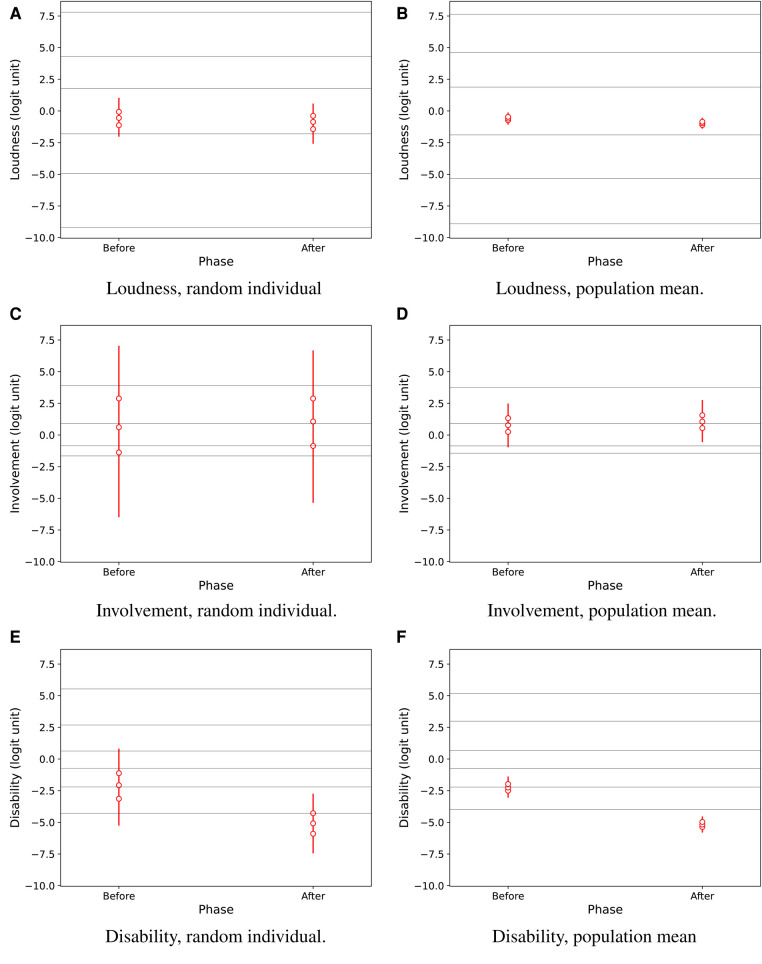
Predictive results for Loudness, Involvement, and Disability for a random individual in the population (**A**,**C**,**E**) and for the population mean (**B**,**D**,**F**), as estimated by regression as a function of the test phase, i.e., before and after hearing-aid fitting. Vertical lines show 95% credible intervals for the marginal distributions of attribute values. Marker symbols show 25-, 50-, and 75-percentiles. Thin horizontal lines across the plots show median estimated response thresholds for the ordinal rating categories.

Figure 4 shows overall effects of the hearing-aid fitting intervention for attributes *Loudness, Involvement*, and *Disability*, in the population from which the participants were recruited. The predicted results for a *random individual* (panels A, C, E) and for the *population mean* (panels B, D, F) suggest a credible effect mainly on *Disability*.

The credibility of intervention effects in the population is reported by *EmaCalc* in separate output tables showing the following results: The probability for a *random individual* was 60.3% for a decrease in *Loudness*, 53.0% for an increase in *Involvement* (i.e., very close to the 50% level indicating no change), and 93.6% for a decrease in *Disability*. The corresponding probability for an intervention effect on the *population mean* was 90.0% for reduced *Loudness*, 59.6% for increased *Involvement*, and higher than 99.9% for a decrease in *Disability*. The very high probability (>99.9%) for improved *Disability* in the population mean can be interpreted in a similar way as a conventional hypothesis test showing a highly significant benefit. The change in *Loudness* had lower probability, and can not be considered clinically relevant anyway, because the change corresponds to only a small fraction within the same ordinal response category (Figure 4B).

**Figure 5:**
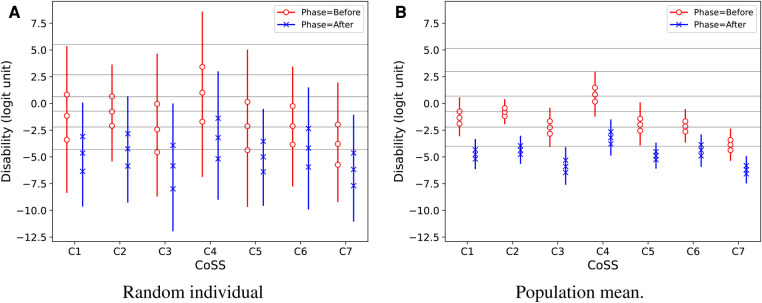
Predictive Disability results for a random individual in the population (**A**) and for the population mean (**B**), as estimated by regression for the combined effects of test phase and CoSS categories. Vertical lines show 95% credible intervals for the marginal distributions for each category. Marker symbols show 25-, 50-, and 75-percentiles. Thin horizontal lines across the plots show median estimated response thresholds for the seven ordinal rating categories.

**Figure 6:**
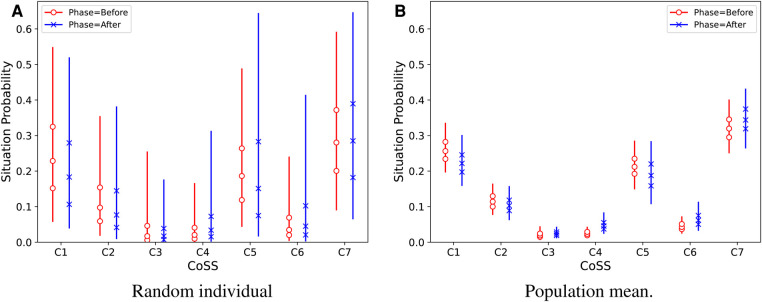
Predictive results for the situation probabilities estimated for a random individual in the population (**A**) and for the population mean (**B**). Vertical lines show 95% credible intervals of the marginal distributions for each category. Marker symbols show 25-, 50-, and 75-percentiles.

Figure 5 displays the predicted intervention effects on *Disability* separately across CoSS categories. The effect is similar in all CoSS categories. The distributions of *situation probabilities* differ markedly by CoSS categories as shown in Figure 6, but there is no obvious effect of the intervention on situation probabilities in any of the CoSS categories.

Figures 4, 5 also show estimated median response thresholds in the population. Thus, the vertical intervals between the thresholds show the latent-variable ranges for each of the ordinal response categories that are defined by their text labels in Figure 3. Therefore, the results can also be interpreted in terms of the raw ordinal response scale: For example, Figure 4E,F shows that the estimated median *Disability* in the population improved from the second or third category, “very slightly disabled” - “slightly disabled,” to the lowest category “not at all disabled.” It is notable that the thresholds are not equally spaced, so the widths of latent-variable response intervals are markedly non-uniform across ordinal response categories, for all three perceptual attributes.

**Figure 7:**
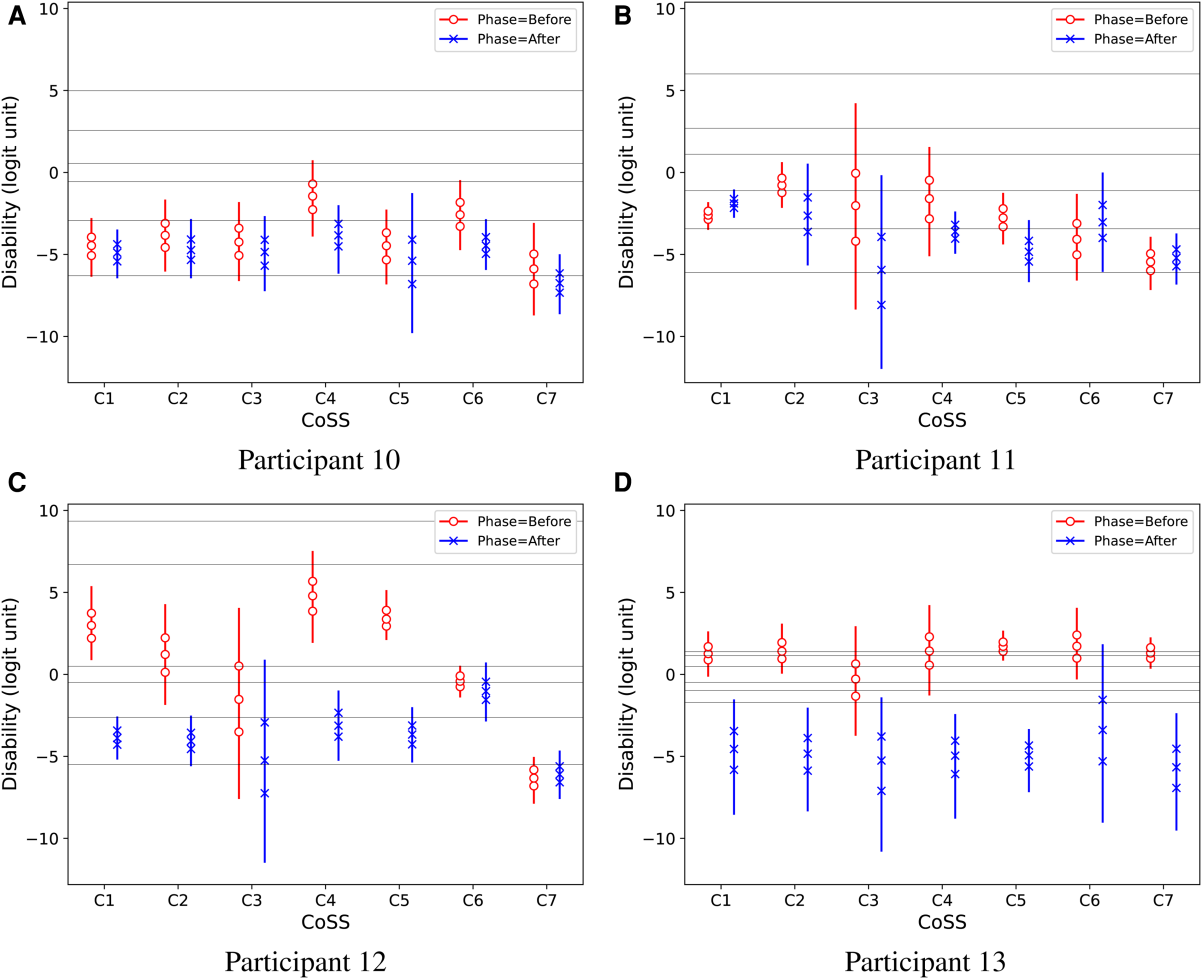
Predictive Disability results of four study participants, denoted by numbers as in the original IHAB-RL study (6, Figures 6, 7). Vertical lines show 95% credible intervals for the marginal distributions of Disability values. Marker symbols show 25-, 50-, and 75-percentiles. Thin horizontal lines across the plots show median estimated response thresholds for the ordinal rating categories. (**A**) Participant 10, (**B**) Participant 11, (**C**) Participant 12 and (**D**) Participant 13.

Figure 7 shows individual intervention effects on *Disability* for four participants denoted with the same numbers as in the original paper (6, Figures 6, 7). Two of these participants showed clearly reduced *Disability* after the intervention, while the other two perceived no clear change. The displayed median response thresholds make it possible to interpret these results also in terms of the raw ordinal response scale: For example, Figure 7C shows that the estimated median intervention effect on *Disability* for participant 12 was three ordinal scale steps in CoSS categories C1, C2, C4, and C5, improving from the fifth category “considerably disabled” to the second category “very slightly disabled.” It is notable that the estimated median response thresholds were markedly different among these participants.

Some participants provided rather scarce EMA data, even no ratings at all in some situations. For example, participants 11 and 12 had no *Disability* ratings at all in CoSS-task C3, neither *Before* nor *After* fitting. In the study phase *After* fitting, participant 10 had no *Disability* ratings at all for CoSS-task C5, and participant 13 did not provide any ratings for CoSS-task C6. Participant 13 provided a total of 44 *Disability* ratings in phase *After*, of which 43 were in the lowest category “not at all disabled.”

The hierarchical Bayesian model (Figure 2) can produce individual estimates even for participants who provided very few ratings in some situations, because the population model acts as a prior distribution for the individual models.^11^ Of course, with a lack of individual data, the uncertainty range of the estimate can get very large, as exemplified in Figure 7, and the results must be interpreted with caution.

## 5. Discussion

### 5.1. Bayesian analysis

The present Bayesian re-analysis of the real EMA data set of von Gablenz et al. (6) revealed an intervention effect on perceived *Disability* with very high statistical credibility for the *population mean* as well as for a *random individual* in the population from which the respondents were recruited. These results are in qualitative agreement with the original analysis results.

The original paper was mainly focused on individual effects, but it also used a frequentist Cumulative Linear Mixed Model (CLMM) regression analysis of data from the same subset of 13 respondents to reveal effects on the group level. The results of von Gablenz et al. (6, Figure 8) showed statistically significant (95% confidence intervals) difference *After* vs. *Before* hearing-aid fitting for *Disability*, a barely significant effect on *Involvement*, and no significant effect on *Loudness*. The regression effect of test phase on the *Disability* ratings amounted to −3.0 (−4.6;−1.5) logit units in the estimated CLMM beta coefficient. The corresponding median improvement was about 2.9 logit units^12^ in the *EmaCalc* model (Figure 4F), but the effect scales are not exactly comparable because *EmaCalc* allows the rating response thresholds to differ between participants.

The CoSS probabilities estimated with the present re-analysis (Figure 6) gave similar results as shown by the distribution of raw EMA responses by CoSS categories in the original paper (6, Figure 4). However, *EmaCalc* predicts the situation probabilities automatically together with their estimated variance in the population.

The *EmaCalc* model allowed the response thresholds to vary between participants, and also allowed non-uniform steps between ordinal response categories on the latent-variable scale. The results in Figures 4, 5 show that the model achieved the best fit to the observed data by using non-uniform steps between response categories. Furthermore, the model found markedly different response thresholds across participants (Figure 7). These results suggest that it might be questionable to presume uniform step sizes between ordinal response categories, and/or to presume that all participants necessarily interpret and use the response alternatives in the same way.

Overall, *EmaCalc* may be an interesting alternative to the CLMM for the statistical analysis of EMA data. The main advantage with the Bayesian method is that it automatically presents measures of the uncertainty of all estimated parameters, both at the participant and the population levels, for the given amount of input data. Therefore, it can predict results both for an unseen *random individual* in the population and for the *population mean*.

However, the *EmaCalc* learning is computationally demanding when many variables are analyzed jointly. The present re-analysis of the IHAB-RL data set used 75 model parameters in total for each participant (2×7=14 for the situation probabilities, 3×14=42 for the regression effects on the three attributes, and 7+5+7=19 for the response thresholds). The variational learning procedure needed 212 iterations to converge, which took about 1.5 h in a MacBookPro (M2, 2022) laptop. The implementation learns model parameters for separate participants in parallel processes, if running in a computer with several CPU cores. Therefore, the computation automatically scales up to handle a large number of participants, limited only by the available hardware.

A known weakness of *EmaCalc* (in its current version) is that it can not use continuous-valued contextual and environmental measures as covariates in the regression model. This may be important for example in EMA studies using data-logging of acoustic sound pressure levels or signal-to-noise ratios (e.g., (28,29)). The extension to continuous-valued metric input data will be considered in a future version.

### 5.2. Alternative frequentist approaches to EMA data analysis

Depending on the context and the objectives for which EMA is used, some frequentist approaches might also serve for analyzing the outcome data. The Cumulative Linear Mixed Model (CLMM), that was used in the original analysis of the IHAB-RL data (6), is theoretically adequate for ordinal EMA data and rather closely related to the Graded Response Model used by *EmaCalc*.

Considering the questionable presumption that raw ordinal scores are metric, von Gablenz et al. (6) used the *Non-overlap of All Pairs (NAP)* (30) as an individual effect measure. The NAP result is a point estimate of the probability that the respondent rates condition B higher than A. Thus, the NAP uses only the ordinal property of the ratings without assuming a metric scale. Since the NAP is a well-defined single-value summary of all rating differences of each participant, it is theoretically a safe aggregation method when testing for differences at population level.

### 5.3. Simplified hypothesis test using mean scores

Researchers might be tempted to use the simplest possible statistical method, at least for a preliminary analysis. Although *“By itself, a p-value does not provide a good measure of evidence regarding a model or hypothesis”* (31), researchers may still consider applying a conventional hypothesis test to the EMA data, rather than the more advanced frequentist CLMM regression model (6) or the Bayesian approach, which provide quantitative effect measures that are more valid as evidence for scientific conclusions.

Since the raw ratings can be considered statistically independent only between different participants, but not within participants, one simple approach (suggested by (8)) is to first somehow aggregate all responses for test conditions A and B separately within each participant, and then apply a standard significance test for a population difference between the two conditions. Although the ratings are correlated within participants, the aggregated intra-individual outcome measures might then safely be analyzed as independent samples from the population of potential respondents.

There are many ways to define a single aggregate measure for each respondent and test condition. Assuming that a researcher primarily wants to compare the difference in *mean score* between two test conditions, Oleson et al. (8) proposed using the average of raw (integer-encoded) ordinal scores as an appropriate aggregate measure. However, when deciding that the mean raw score is the most relevant quantity to be compared, the researcher must already presume that the raw scores represent values on an interval scale, and Oleson et al. (8) also noted that it may be difficult to address the question whether the distances between ordinal categories are actually uniform.

The original analysis of the IHAB-RL data presented mean and standard deviations of raw scores for individual ratings (6, Figure 6), although the authors also pointed to the questionable assumption of metric raw data. Anyway, let us now temporarily disregard these fundamental scale problems, and apply the mean-score aggregation method also for a test on the population level. In this case we cannot assume that the EMA data are normally distributed: A Shapiro-Wilks test indicated significant non-normality (p=0.009) for *Disability*, and the *Involvement* data are too scarce for relying on the central limit theorem. Only the data for *Loudness* might be a candidate for a test that requires normal distribution. Therefore, the mean scores were instead submitted to a two-tailed Wilcoxon signed-rank test (scipy.stats.wilcoxon). The results indicated a highly significant effect of hearing-aid uptake for *Disability* (T=1.0;N=13;p<0.001) but no significant effect on *Involvement* (T=17.0;N=12;p=0.155) and *Loudness* (T=30.0;N=13;p=0.305), again qualitatively in agreement with the *EmaCalc* results for the population mean.

We think statistical tests using the mean scores should be used with caution, although they may lead to similar conclusions as a theoretically safer CLMM regression analysis or a Bayesian approach.

## 6. Conclusions

As suggested in a recent tutorial about the statistical challenges of EMA data (8), a hierarchical Bayesian latent-variable analysis model has been developed and made freely available to researchers. The *EmaCalc* implementation in Python can handle EMA input data including nominal situation categories and ordinal ratings of perceptual attributes, and can estimate the statistical relation between these variables.

Although researchers may be tempted to use the simplest possible “mean-score” methods, those methods presume that ordinal response categories represent uniform steps on a perceptual scale, and this presumption may be questionable and difficult to motivate.

The proposed latent-variable model avoids this fundamental presumption by allowing non-uniform step sizes between ordinal categories on the response scale. The model also allows response thresholds to vary between participants, so it does not presume that all participants necessarily interpret and use the ordinal categories in the same way.

The new model was used to re-analyze an EMA data set (6) including 13 first-time hearing-aid users reporting about listening situations and subjective impressions in two study phases: before and after hearing-aid fitting. The hierarchical model estimated intervention effects in the population from which the participants were recruited. The results for the *population mean* were similar to those obtained in the previous analysis by an advanced frequentist ordinal regression model.

The Bayesian approach automatically estimated the inter-individual variability in the population, based on the study sample, and could show some statistically credible intervention results also for an *unseen random individual* in the population. Such results may be interesting, for example, if the EMA methodology is used by a hearing-aid manufacturer in a study to predict the success of a new signal-processing method among future potential customers.

## Data Availability

The data analyzed in this study is subject to the following licenses/restrictions: The analysed IHAB-RL data set can be accessed by reasonable request directed to Petra von Gablenz, petra.vongablenz@jade-hs.de.

## Footnotes

^1^If separate groups of participants are recruited from different populations, the number of respondents N may, of course, differ among groups. The analysis model has the same structure for each group, so the same math notation can refer to any of the groups/populations.

^2^In IRT, the thresholds are usually assumed identical across respondents, and may even be further restricted to reduce the number of free parameters (e.g., (17)). In EMA we typically have many records from each respondent, with ratings ideally spanning the entire range of ordinal grades, so the amount of data is usually sufficient to estimate individual thresholds.

^3^*EmaCalc* can be set up to use the same response thresholds for more than one attribute question.

^4^The Cumulative Model can also use the normal distribution.

^5^The unity scale is no restriction of generality, as the model scale is arbitrary anyway.

^6^The implemented model can also use the “ordinal-probit” variant (Thurstone Case V) assuming normal (Gaussian) distribution for the latent variables.

^7^Random variables can be statistically dependent even if their correlation is zero.

^8^Data files are accessed by sub-package *pandas* that can handle several table-style file formats, such as xlsx, csv. Recorded data may be converted to agree with variable names and labels defined in the setup step.

^9^Figures can be saved in any format allowed by Python sub-package *matplotlib*, such as eps, pdf, png. Tabular results can be saved in any format that sub-package *pandas* allows, such as txt, tex, xlsx, csv.

^10^CoSS specifies situational intentions and tasks by seven main categories: “Speech conversation, two persons,” “Speech conversation, more than two persons,” “Speech by communication device,” “Focused listening (live),” “Focused listening (media),” “Monitoring surroundings,” and “Passive listening.”

^11^A similar effect can be achieved by so-called “partial pooling” in conventional frequentist GLM estimation.

^12^The “logit unit” in *EmaCalc* is the standard deviation of a logistic distribution with unity scale, which corresponds to the logit link function used in the CLMM.
