# Supplementary material for: Bayesian analysis of Ecological Momentary Assessment (EMA) data collected in adults before and after hearing rehabilitation
Source: Front Digit Health. 2023 Feb 17;5:1100705. doi: 10.3389/fdgth.2023.1100705 (PMC9981641; doi:10.3389/fdgth.2023.1100705)
Supplement: Supplementary file 1 [file Presentation1.pdf]

EmaCalc Math Details

February 3, 2023

This report defines all mathematical details of the proposed method for Bayesian analysis of EMA data, thus supplementing the overview of the theory presented in the associated main paper.

A Individual Model Parameters

As outlined in section 3.2 of the main paper, all nominal and ordinal response patterns from the nth participant are assumed probabilistically determined by a parameter vector $\boldsymbol{\xi}_n = (\ldots, \boldsymbol{\alpha}_{nt}, \ldots, \boldsymbol{\beta}_{ni}, \ldots, \boldsymbol{\eta}_n, \ldots)$ with three classes of parameters: The $\boldsymbol{\alpha}_{nt}$ parameter subset define the situation probabilities, $\boldsymbol{\beta}_{ni}$ are the situation effects on the ith attribute value, and the elements of $\boldsymbol{\eta}_n$ define the response thresholds. The conditional log-likelihood of all responses, given the parameters, are defined mathematically in the following sections A.1, A.2, and A.3.

In the following, the elements of this parameter vector are denoted either as ξ_{nd} or, equivalently, by the separate symbols α, β, η for the three classes of parameters. The total number of parameters, and the indexing of parameter sub-classes, depends on the experimental structure and the regression effects to be estimated, but this notational equivalence is defined once and for all when the analysis model is set up.

A.1 Nominal EMA Situation Responses

Based on the rth recorded EMA response by the nth participant in the tth test phase, the situation is identified by a so-called 1-of-K binary vector $\boldsymbol{z}_{nrt}$ with one element $z_{nrt,k} = 1$ indicating that the kth situation category was selected, and all other $z_{nrt,j \neq k} = 0$.

If the study includes several situation dimensions identifying, e.g., the participant's listening task or the background noise level, the situation response from each participant might also be indexed as $z_{nrt,k_1,k_2 \ldots}$ where k_1

is the index identifying the category in the first situation dimension, and k_2 is the index in the second dimension, etc. The multi-dimensional index $(k_1, k_2, \ldots)$ can always be uniquely represented by an equivalent linear index k, and vice versa. Therefore, we use mainly the one-dimensional index notation in the mathematical formulation.

The model assumes that the occurrence of various situations in the EMA records are determined by fixed but unknown *situation probability* vectors $\boldsymbol{u}_{nt} = (u_{nt1}, \ldots, u_{ntK})$. Here, $u_{ntk} \in [0, 1]$ is the probability that the nth participant reports from the kth situation category at any assessment in the tth test phase. The array of situation probabilities is one of the main results to be estimated from the data.

The individual situation probabilities are assumed to be the same for all EMA records from the same test phase, but may vary between participants and between test phases. The recorded EMA situation responses are assumed conditionally independent, given the parameters. Thus, the responses follow a categorical distribution, conditional on the probabilities,

$$p(\boldsymbol{z}_{nrt} \mid \boldsymbol{u}_{nt}) = \prod_{k=1}^{K} u_{ntk}^{z_{nrt,k}}; \quad \sum_{k=1}^{K} u_{ntk} = 1; \quad \forall n, r, t. \tag{A.1}$$

To ensure that $\boldsymbol{u}_{nt}$ is restricted to be a properly normalized probability-mass vector during the computation, each vector is mapped as a function of a parameter vector $\boldsymbol{\alpha}_{nt} = (\alpha_{nt1}, \ldots, \alpha_{ntK})$ with unrestricted real-valued elements, by the softmax function

$$u_{ntk}(\boldsymbol{\xi}_n) = \frac{e^{\alpha_{ntk}}}{\sum_{j=1}^{K} e^{\alpha_{ntj}}}, \quad \forall n, t, k. \tag{A.2}$$

All parameters α_{ntk} for the nth participant are stored as the TK first elements in the individual parameter vector $\boldsymbol{\xi}_n$. Denoting the total array of all situation responses as $\underline{\boldsymbol{z}}_n = (\ldots, \boldsymbol{z}_{nrt}, \ldots)$, the log-likelihood of all observed nominal situation data for the nth participant is

$$\ln p(\underline{\boldsymbol{z}}_n \mid \boldsymbol{\xi}_n) = \sum_{r=1}^{R_n} \sum_{t=1}^{T} \sum_{k=1}^{K} z_{nrt,k} \ln u_{ntk}(\boldsymbol{\xi}_n). \tag{A.3}$$

A.2 Ordinal EMA Attribute Ratings

Based on the ordinal rating to the ith attribute question (with L_i ordinal response alternatives), the response is identified by a 1-of-L_i binary vector $\boldsymbol{y}_{nri}$ with one element $y_{nri,l} = 1$ indicating that the nth participant gave the lth ordinal response to the ith question in the rth assessment, and all other

$y_{nri,j \neq l} = 0$. The rth record also specifies the current situation by the multi-dimensional situation index vector $\boldsymbol{k}(r) = (k_0, k_1, \ldots)(r)$. The first index identifies the test phase $k_0 = t$ which is defined by the researcher, and the other indices $(k_1, k_2, \ldots)$ specify the situation as identified by the respondent, as defined in the previous section. The multi-dimensional array index $\boldsymbol{k}(r)$ can always be uniquely represented by an equivalent scalar linear index $k(r)$, so this notation is used where it is unambiguous.

As discussed in section 3.2.3 in the main paper, the ordinal rating responses are analyzed with a variant of Item Response Theory (IRT). In the model, illustrated in Figure 1, each ordinal response is determined by an outcome of a continuous real-valued latent random variable $Y_{nik(r)}$. The lth ordinal response is given whenever the latent variable falls in an interval $\tau_{ni,l-1} < Y_{nik(r)} \leq \tau_{ni,l}$. where the thresholds separating the intervals form an increasing sequence $(-\infty = \tau_{ni,0} < \tau_{ni,1}, \ldots, < \tau_{ni,L_i} = +\infty)$. The latent variable is drawn from a logistic probability distribution with location θ_{nik} and unity scale.

The location parameters $(\ldots, \theta_{nik}, \ldots)$ for the nth participant include, in principle, a large number of free parameters, one for each combination of nominal categories from all situation dimensions. However, just as in linear regression, the model may be simplified to consider only a linear combination of a smaller set of *situation effects*, e.g., by expressing the attribute location value as $\theta_{nik_0k_1k_2} = \beta_{nik_0} + \beta_{nik_1} + \beta_{nik_2}$, or $\theta_{nik_0k_1k_2} = \beta_{nik_0k_1} + \beta_{nik_2}$. Here, the value β_{nik_f} represents the *main effect* of the k_fth category in the fth situation dimension, while $\beta_{nik_0k_1}$ also represents *interaction effects* between any combination (k_0, k_1) of categories in the zeroth and first situation dimensions. Regardless of these indexing details, the location parameter can always be specified as a fixed linear function $\theta_{nik}(\boldsymbol{\beta}_{ni})$ of an effect vector $\boldsymbol{\beta}_{ni} = (\ldots, \beta_{nij}, \ldots)$ including all the user-selected effects to be estimated by the regression model.

All threshold values are determined by a mapping function $\tau_{ni,l}(\boldsymbol{\eta}_n)$ defined in section A.3 such as to ensure that the response thresholds form a strictly increasing sequence for each item. If desired, the mapping can also be specified to yield identical thresholds across different items. Thus, the array $\boldsymbol{\eta}_n$ includes parameters defining all response thresholds for the nth participant.

From now on, we denote the individual ordinal-model parameters $\boldsymbol{\beta}_n, \boldsymbol{\eta}_n$ as parts of the total individual parameter vector $\boldsymbol{\xi}_n$, with an indexing scheme determined by the structure of the model and the desired analysis results.

Finally, using this simplified parameter notation, the conditional probability of any rating response, given the model parameters, is a known function

of the parameters,

$$\begin{aligned} &\mathsf{P}(y_{nri,l} = 1 \mid \boldsymbol{\xi}_n) = \mathsf{P}(\tau_{ni,l-1} < Y_{ni\,k(r)} \leq \tau_{ni,l} \mid \boldsymbol{\beta}_{ni}) = \\ &\quad = F\left(\tau_{ni,l} - \theta_{nik(r)}\right) - F\left(\tau_{ni,l-1} - \theta_{nik(r)}\right) \stackrel{\text{def.}}{=} v_{ik(r),l}(\boldsymbol{\xi}_n), \quad \forall n, r, i, \end{aligned} \tag{A.4}$$

where $F(\ )$ is the cumulative distribution function for a standard logistic random variable,

$$F(x) = \frac{1}{1 + e^{-x}}. \tag{A.5}$$

All ratings, gathered in an array $\underline{\boldsymbol{y}}_n = (\ldots, \boldsymbol{y}_{nri}, \ldots)$, are assumed conditionally independent across assessments and questions, given the individual parameters $\boldsymbol{\xi}_n$. Thus the log-likelihood of the observed ordinal response data from the nth participant is

$$\ln p(\underline{\boldsymbol{y}}_n \mid \boldsymbol{\xi}_n) = \sum_{r=1}^{R_n} \sum_{i=1}^{I} \sum_{l=1}^{L_i} y_{nri,l} \ln v_{ik(r),l}(\boldsymbol{\xi}_n). \tag{A.6}$$

Since this regression model allows both the perceptual attribute value θ_{nik} and the threshold parameters $\tau_{ni,l}$ to be freely variable for each respondent, the model is under-determined: If a fixed constant value is added to all θ_{nik} and all $\tau_{ni,l}$, the probability (A.4) of observed responses does not change. A weakly informative prior distribution, defined in Section B below, slightly favors solutions with parameters near zero, so the learning always converges. However, the indeterminacy allows some artificial variability in the parameter values. To avoid this variance, the parameter values must be somehow restricted. The current implementation allows the researcher either (1, default) to force the median response threshold to zero, or (2) to force the average attribute value to zero, for each respondent and each attribute.

A.3 Ordinal Response Thresholds

To ensure that the response thresholds form a strictly increasing sequence, it is convenient to define each response-interval width $\tau_{ni,l} - \tau_{ni,l-1}$ by its corresponding width $F(\tau_{nil}) - F(\tau_{ni,l-1})$ in the range $[0, 1]$, using a mapping function

$$F(\tau_{nil}) = \frac{\sum_{j=1}^{l} w(\eta_{nij})}{\sum_{j=1}^{L_i} w(\eta_{nij})}, \tag{A.7}$$

where $F(\ )$ is the logistic distribution function defined in (A.5), and the $w(\eta_{nij})$ are relative interval widths. The widths are mapped from a parameter vector[1] $\boldsymbol{\eta}_{ni} = (\eta_{ni1}, \ldots, \eta_{nil}, \ldots, \eta_{niL_i})$ with elements in the full range

[1]Thresholds for several items may be mapped from the same $\boldsymbol{\eta}$ array, although this is not reflected in the notation here.

$(-\infty, +\infty)$. The inverse mapping defines the thresholds as

$$\tau_{ni,l}(\boldsymbol{\eta}_{ni}) = \ln \frac{\sum_{j=1}^{l} w(\eta_{nij})}{\sum_{j=l+1}^{L_i} w(\eta_{nij})}. \tag{A.8}$$

Scaling all widths $w(\eta_{nij})$ by any positive factor does not change the resulting thresholds, so the L_i parameter values actually define only $L_i - 1$ free thresholds, as desired.

The width-mapping function must be monotonously increasing and differentiable, but is otherwise not critical. It is chosen for numerical convenience as

$$w(\eta) = e^{\eta} + \epsilon, \tag{A.9}$$

where ϵ is a tiny positive constant preventing numerical underflow in the computation.

B Population model

All nominal and ordinal response patterns from the nth participant were assumed determined by a parameter array $\boldsymbol{\xi}_n = (\xi_{n1}, \ldots, \xi_{nd}, \ldots, \xi_{nD})$ including three separate classes of parameters, as defined in section A above.

As all participants were recruited at random from the same population, as defined by the researcher, the model treats each individual parameter vector $\boldsymbol{\xi}_n$ as a sample drawn at random from a population distribution.

B.1 Gaussian mixture model

The individual parameter vectors are assumed drawn from a population distribution in the form of a Gaussian mixture model (GMM),

$$p(\boldsymbol{\xi}_n \mid \underline{\boldsymbol{\mu}}, \underline{\boldsymbol{\lambda}}, \underline{\boldsymbol{\zeta}}) = \prod_{c=1}^{C} \left[\prod_{d=1}^{D} \sqrt{\frac{\lambda_{cd}}{2\pi}} e^{-(\xi_{nd} - \mu_{cd})^2 \lambda_{cd}/2} \right]^{\zeta_{nc}}, \quad \forall n, \tag{B.1}$$

where $\boldsymbol{\mu}_c = (\mu_{c,1}, \ldots, \mu_{c,D})$ is the mean vector, and $\boldsymbol{\lambda}_c = (\lambda_{c,1}, \ldots, \lambda_{c,D})$ is the precision vector (inverse variance) of the cth mixture component, with $\underline{\boldsymbol{\mu}} = (\boldsymbol{\mu}_1, \ldots, \boldsymbol{\mu}_C)$ and $\underline{\boldsymbol{\lambda}} = (\boldsymbol{\lambda}_1, \ldots, \boldsymbol{\lambda}_C)$ denoting the total set of parameters for all mixture components (in each population model). The selected mixture component is indicated by a latent 1-of-C binary array $\boldsymbol{\zeta}_n = (\zeta_{n1}, \ldots, \zeta_{nC})$, where $\zeta_{nc} = 1$ indicates that the nth respondent has a parameter vector drawn from the cth mixture component, and all other elements are $\zeta_{n,j \neq c} = 0$.

It is no serious restriction to assume independent elements (diagonal covariance) in each mixture component, because the complete mixture model can still capture any statistical dependence between elements of $\boldsymbol{\xi}_n$. If several subject groups are included, representing different populations, a separate GMM is defined for each population, although this is not reflected in the math notation here. The number of mixture components may differ among population models.

Of course, it is never known exactly from which mixture component the individual parameter vector $\boldsymbol{\xi}_n$ is actually generated. Therefore, the resulting mixture density can be equivalently written as a weighted sum across all mixture components,

$$p(\boldsymbol{\xi}_n \mid \underline{\boldsymbol{\mu}}, \underline{\boldsymbol{\lambda}}) = \sum_{c=1}^{C} \langle \zeta_{nc} \rangle \prod_{d=1}^{D} \sqrt{\frac{\lambda_{cd}}{2\pi}} e^{-\frac{1}{2}(\xi_{nd} - \mu_{cd})^2 \lambda_{cd}}. \tag{B.2}$$

with weights equal to the means $\langle \zeta_{nc} \rangle = \mathsf{E}[\zeta_{nc}] \in (0, 1)$.

In the Bayesian framework, all these parameters are again modeled as random variables with weakly informative prior distributions reflecting the prior knowledge and assumptions about the model.

B.2 GMM parameter priors

A single Gauss-gamma prior is defined for all GMM components in all population models:

$$p(\underline{\boldsymbol{\mu}}, \underline{\boldsymbol{\lambda}}) = \prod_{c=1}^{C} p(\boldsymbol{\mu}_c, \boldsymbol{\lambda}_c); \tag{B.3}$$

$$p(\boldsymbol{\mu}_c, \boldsymbol{\lambda}_c) = \prod_{d=1}^{D} p(\mu_{cd} \mid \lambda_{cd}) p(\lambda_{cd}); \tag{B.4}$$

$$p(\mu_{cd} \mid \lambda_{cd}) = \sqrt{\frac{\nu' \lambda_{cd}}{2\pi}} e^{-\frac{1}{2}(\mu_{cd} - m'_{cd})^2 \nu' \lambda_{cd}}; \tag{B.5}$$

$$p(\lambda_{cd}) = \frac{{b'_d}^{a'}}{\Gamma(a')} \lambda_{cd}^{a'-1} e^{-b'_d \lambda_{cd}}. \tag{B.6}$$

Here, $\Gamma(z) = \int_0^\infty x^{z-1} e^{-x}\, dx$ is the gamma function. In the absence of prior information, we assign all $m'_{cd} = 0$, except for the log-probabilities α that are set for a uniform distribution of situation probabilities. The Jeffreys prior for the mean and precision of a Gaussian distribution would suggest $\nu' \to 0, a' \to 0, b'_d \to 0$. However, to avoid computational indeterminacy causing numerical overflow in case of extreme response patterns, e.g., if all respondents use only the highest ordinal rating, we must use a weakly informative prior.

The effective weight of the prior on the population mean, relative to the weight of one real test participant, is assigned as $\nu' = 0.5$. The prior gamma-distribution shape parameter is assigned as $a' = \nu'/2$, and the inverse scale $b'_d = \sigma_d^2/2$, where σ_d is a crude estimate of the magnitude of parameter variations, as specified below. The factor 1/2 is included to conform with the update equations (C.24) and (C.25).

Setting b'_d clearly greater than zero can have an effect on the *Occam's Razor* automatic determination of model complexity, because it prevents the Gaussian components from becoming centered on a single data point with infinite precision (zero variance). With any $a' < 1$ the prior probability density for the precision λ_{cd} is concentrated near zero. The prior expectation of the variance $1/\lambda_{cd}$ is undefined for $a' < 1$, but the mode is

$$\text{mode}\left[1/\lambda_{cd} \mid a', b'_d\right] = \frac{b'_d}{a' + 1} \approx b'_d. \tag{B.7}$$

The prior scale of parameters ξ_d is assigned as $\sigma_d = 1$ for all elements related to the logarithmic parameters α_{nt} for the nominal situation probabilities in (A.3). The same prior scale is also assigned for all elements related to the logarithmic parameters η_{nl} for the rating thresholds in (A.8) and (A.6). For all elements related to the effect parameters β_{nij} in (A.6), the scale is $\sigma_d = \pi/\sqrt{3}$ = the standard deviation of a unity-scale logistic distribution. The prior parameter distributions with these hyperparameter settings are broad with fat tails, as illustrated in Figs. B.1a and B.1b. The selected values are specified as global variables in module `ema_base.py`, so users can easily experiment with other settings.

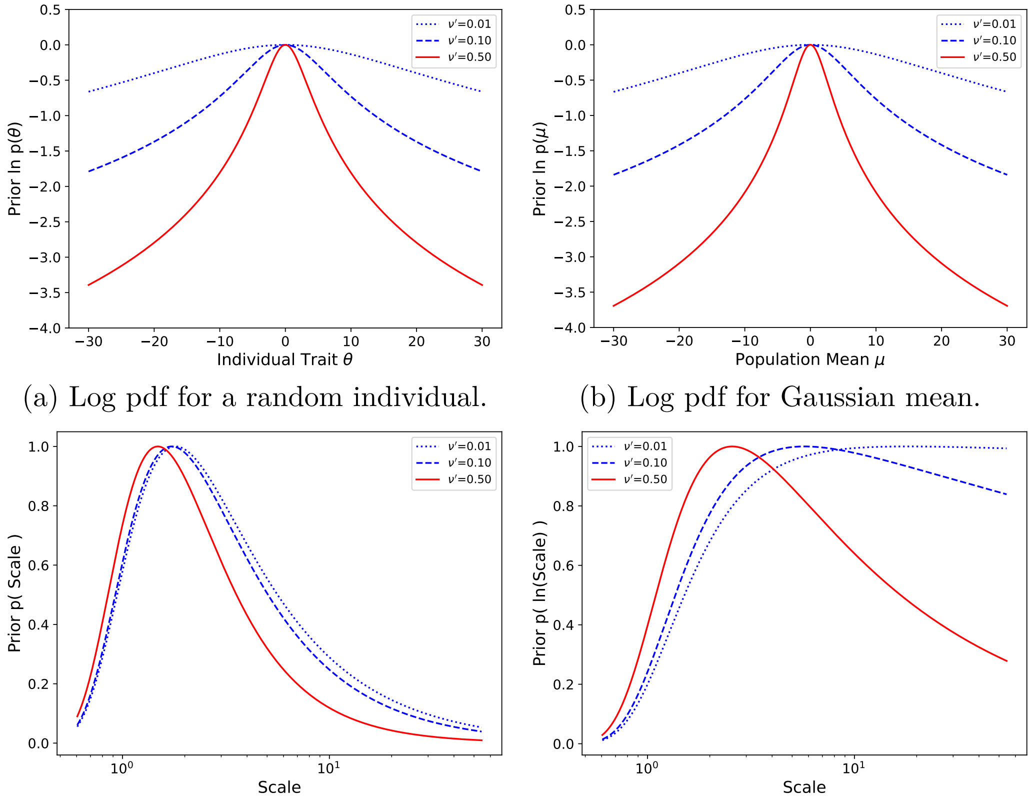

(a) Log pdf for a random individual.

(b) Log pdf for Gaussian mean.

(c) Pdf for Gaussian scale.

(d) Pdf for Gaussian log-scale.

Figure B.1: Marginal prior log pdfs for (a) an attribute value θ of a random individual, and (b) for the mean μ_{cd}, and (c) pdf for the scale s, and (d) log-scale $\ln(s)$, with $s = \sqrt{1/\lambda_{cd}}$, with the population Gaussian densities $p(\mu_{cd}, \lambda_{cd})$ in (B.4). The plot shows density functions for any dth element related to attribute regression-effect parameters, with hyperparameters $\nu' = 0.5$ and two other values, $a' = \nu'/2$, and $\sigma_d = \pi/\sqrt{3}$, as discussed in Sec. B.2. The marginal distributions for the attribute value and the mean are Student-t as shown in (D.2) and (D.1). The distribution of the variance $1/\lambda_{cd}$ is inverse-gamma, which has been transformed here to show the pdf for s and $\ln(s)$, plotted as a function of s on the logarithmic horizontal axis. For a completely non-informative (improper) prior, the densities for the mean and the log-scale would be uniform, but the present prior was deliberately designed as slightly informative to discourage very large means and very small scales. The density functions have been re-scaled to the same maximum, i.e., they are not normalized.

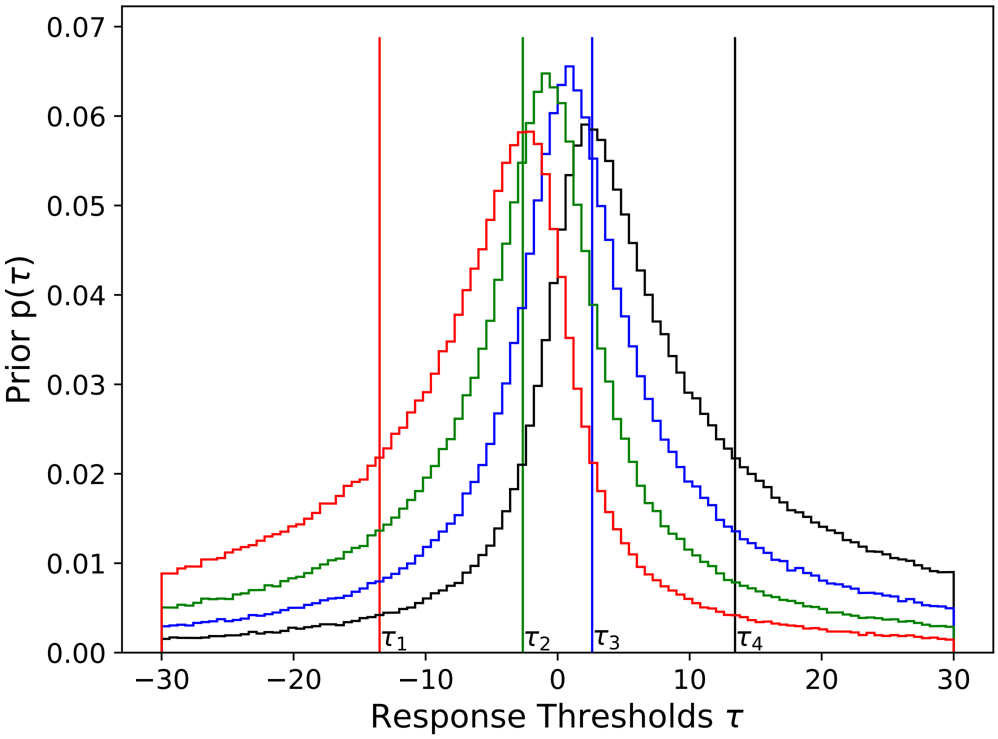

Figure B.2: Marginal prior pdfs of the four free response thresholds of a random individual, for an attribute item with five ordinal response alternatives. The ragged color-marked curves show density functions approximated by histograms of 1 million samples, with hyperparameters $\nu' = 0.5$, $a' = \nu'/2$, and $\sigma_d = 1$, as discussed in Sec. B.2. Vertical lines with matching colors show medians of the threshold distributions.

B.3 Mixture weights

The prior mixture weights are denoted $\boldsymbol{v} = (v_1, \ldots, v_C)$, where element v_c is the probability that any random individual in the modeled population has a parameter vector drawn from the cth mixture component, i.e.,

$$\mathsf{P}(\zeta_{nc} = 1 \mid \boldsymbol{v}) = v_c, \quad \forall n. \tag{B.8}$$

The prior distribution of mixture weights $\boldsymbol{v}$ is assigned as a Dirichlet distribution with concentration parameters $\boldsymbol{\gamma}' = (\gamma'_1, \ldots, \gamma'_C)$,

$$p(\boldsymbol{v}) = \frac{1}{B(\boldsymbol{\gamma}')} \prod_{c=1}^{C} v_c^{\gamma'_c - 1}. \tag{B.9}$$

Here, the normalizing $B(\;)$ is the multivariate Beta function. We follow the conventional approach for training Bayesian mixture models and assign fixed small concentration parameters with equal small values for all c in order for the learning to favor sparse solutions. The implementation uses the Jeffreys prior $\gamma'_c = 0.5$ for all c.

This prior value plays the role of pseudo-counts in the estimation of mixture weights $\boldsymbol{v}$. Even if none of the study participants is related to, say, the cth mixture component, the variational distribution of mixture weights for a random individual will in effect be calculated as if 0.5 subject had actually had a response pattern related to this component. The use of the Jeffreys prior as pseudo-count is a theoretically well-defined way to keep the learning procedure open for the possibility that the next subject that could have been recruited from the same population might actually be characterized by response-model parameters drawn from the cth component.

C Model learning

C.1 Total log-likelihood

We now denote the array of all ordinal rating data across subjects as $\underline{\boldsymbol{y}} = (\ldots, \boldsymbol{y}_n, \ldots)$, and all nominal situation data as $\underline{\boldsymbol{z}} = (\ldots, \boldsymbol{z}_n, \ldots)$. The corresponding array of all individual parameters is written $\underline{\boldsymbol{\xi}} = (\ldots, \boldsymbol{\xi}_n, \ldots)$. The observations are considered conditionally independent across subjects, given the parameters, so the total log-likelihood is just a sum across individual log-likelihood functions.

Using the conditional log-likelihood for individual data in (A.3) and (A.6), together with the prior parameter densities in (B.1), (B.5), (B.6) and (B.9),

the total log-likelihood of all observed data and all model[2] parameters is[3] (omitting irrelevant constants)

$$\begin{aligned}\ln p(\underline{\boldsymbol{y}}, \underline{\boldsymbol{z}}, \underline{\boldsymbol{\xi}}, \underline{\boldsymbol{\mu}}, \underline{\boldsymbol{\lambda}}, \underline{\boldsymbol{\zeta}}, \boldsymbol{v}) = \text{const.} + \sum_{n=0}^{N-1} \ln p(\boldsymbol{y}_n \mid \boldsymbol{\xi}_n) + \ln p(\boldsymbol{z}_n \mid \boldsymbol{\xi}_n) \\ + \sum_{n=0}^{N-1} \sum_{c=1}^{C} \zeta_{nc} \sum_{d=1}^{D} \frac{1}{2} \ln \lambda_{cd} - \frac{1}{2} (\xi_{nd} - \mu_{cd})^2 \lambda_{cd} \\ + \sum_{c=1}^{C} \sum_{d=1}^{D} \frac{1}{2} \ln \lambda_{cd} - \frac{1}{2} (\mu_{cd} - m'_{cd})^2 \nu' \lambda_{cd} + (a' - 1) \ln \lambda_{cd} - b'_d \lambda_{cd} \\ + \sum_{c=1}^{C} (\gamma'_c - 1) \ln v_c + \sum_{n=0}^{N-1} \zeta_{nc} \ln v_c \end{aligned} \quad \text{(C.1)}$$

Here the first line represents the log-likelihood of observed nominal and ordinal EMA data, given the individual model parameters, the second line is the log probability density of individual model parameters as samples from the population GMM, and the remaining two lines specify the prior log probability density for the population-model parameters.

C.2 Variational inference

The model is trained from data with a variant of the standard Variational Inference (VI) procedure (e.g., Bishop, 2006, Ch. 10). A partially factorized density function $q(\ )$ is adapted to be a good approximation of the exact posterior density $p(\ )$ of all model parameters, given the observed data, as

$$p(\underline{\boldsymbol{\xi}}, \underline{\boldsymbol{\mu}}, \underline{\boldsymbol{\lambda}}, \underline{\boldsymbol{\zeta}}, \boldsymbol{v} \mid \underline{\boldsymbol{y}}, \underline{\boldsymbol{z}}) \approx q(\underline{\boldsymbol{\xi}}, \underline{\boldsymbol{\mu}}, \underline{\boldsymbol{\lambda}}, \underline{\boldsymbol{\zeta}}, \boldsymbol{v}) = q(\underline{\boldsymbol{\xi}}, \underline{\boldsymbol{\zeta}}) q(\underline{\boldsymbol{\mu}}, \underline{\boldsymbol{\lambda}}) q(\boldsymbol{v}). \quad \text{(C.2)}$$

This variational approximation factorizes the density for the total set of model parameters between the individual parameters $q(\underline{\boldsymbol{\xi}}, \underline{\boldsymbol{\zeta}})$, the set of Gaussian component parameters $q(\underline{\boldsymbol{\mu}}, \underline{\boldsymbol{\lambda}})$, and the component probabilities $q(\boldsymbol{v})$.

The VI procedure maximizes a lower bound to the data log-likelihood[4],

$$\mathcal{L}(q) = \left\langle \ln \frac{p(\underline{\boldsymbol{y}}, \underline{\boldsymbol{z}}, \underline{\boldsymbol{\xi}}, \underline{\boldsymbol{\mu}}, \underline{\boldsymbol{\lambda}}, \underline{\boldsymbol{\zeta}}, \boldsymbol{v})}{q(\underline{\boldsymbol{\xi}}, \underline{\boldsymbol{\mu}}, \underline{\boldsymbol{\lambda}}, \underline{\boldsymbol{\zeta}}, \boldsymbol{v})} \right\rangle_{q()} \leq \ln p(\underline{\boldsymbol{y}}, \underline{\boldsymbol{z}}) \quad \text{(C.3)}$$

[2]The same notation applies to each group / population model. The total log-likelihood is just a sum across group models, if there is more than one.

[3]The summation signs on each line refers to all terms only on the same line, not other lines.

[4]The notation $\langle\ \rangle_{q(\ )}$ means expectation calculated using the current $q(\ )$ distribution

and minimizes the Kullback-Leibler divergence

$$\mathrm{KL}\,(q\,\|\,p) = \left\langle \ln \frac{q(\underline{\boldsymbol{\xi}}, \underline{\boldsymbol{\mu}}, \underline{\boldsymbol{\lambda}}, \underline{\boldsymbol{\zeta}}, \boldsymbol{v})}{p(\underline{\boldsymbol{\xi}}, \underline{\boldsymbol{\mu}}, \underline{\boldsymbol{\lambda}}, \underline{\boldsymbol{\zeta}}, \boldsymbol{v} \mid \underline{\boldsymbol{y}}, \underline{\boldsymbol{z}})} \right\rangle_{q(\;)} \tag{C.4}$$

between the approximate and exact posterior parameter distributions. The procedure is iterative and theoretically guaranteed to converge.

Since (C.1) includes only a sum of terms across n for the individual parameters, and only a sum across c for the mixture components, while the individual and the population parameters are linked only by the latent indicators $\underline{\boldsymbol{\zeta}}$, the variational distributions are naturally factorized without any further approximation, as

$$q(\underline{\boldsymbol{\xi}}, \underline{\boldsymbol{\zeta}}) = \prod_{n=0}^{N-1} q(\boldsymbol{\xi}_n, \boldsymbol{\zeta}_n) = \prod_{n=0}^{N-1} q(\boldsymbol{\zeta}_n \mid \boldsymbol{\xi}_n) q(\boldsymbol{\xi}_n) \tag{C.5}$$

$$q(\underline{\boldsymbol{\mu}}, \underline{\boldsymbol{\lambda}}) = \prod_{c=1}^{C} q(\boldsymbol{\mu}_c, \boldsymbol{\lambda}_c) = \prod_{c=1}^{C} \prod_{d=1}^{D} q(\mu_{cd} \mid \lambda_{cd}) q(\lambda_{cd}) \tag{C.6}$$

Since the prior Gauss-gamma are conjugate distributions for the parameters of Gaussian mixture components, the variational $q(\mu_{cd}, \lambda_{cd})$ will naturally get the same Gauss-gamma form as the priors, without any further approximation. Similarly, the variational $q(\boldsymbol{v})$ will also naturally become a Dirichlet density. However, the individual parameter distributions $q(\boldsymbol{\xi}_n)$ cannot be expressed as a member of a known distribution family. Therefore, the parameter density $q(\boldsymbol{\xi}_n)$ is approximated by a large number of equally probable sample vectors $\hat{\boldsymbol{\xi}}_{ns} = (\ldots, \hat{\xi}_{nsd}, \ldots)$, generated by Hamiltonian sampling (Neal, 2011).

C.2.1 Individual variables

The variational distribution $q(\underline{\boldsymbol{\xi}}, \underline{\boldsymbol{\zeta}})$ is obtained by averaging (C.1) across the distributions of other parameters, as

$$\ln q(\underline{\boldsymbol{\xi}}, \underline{\boldsymbol{\zeta}}) = \left\langle \ln p(\underline{\boldsymbol{y}}, \underline{\boldsymbol{z}}, \underline{\boldsymbol{\xi}}, \underline{\boldsymbol{\mu}}, \underline{\boldsymbol{\lambda}}, \underline{\boldsymbol{\zeta}}, \boldsymbol{v}) \right\rangle_{q(\underline{\boldsymbol{\mu}}, \underline{\boldsymbol{\lambda}}, \boldsymbol{v})} + \text{const.} = \sum_{n=0}^{N-1} \ln q(\boldsymbol{\xi}_n, \boldsymbol{\zeta}_n) \tag{C.7}$$

yielding

$$\begin{aligned} \ln q(\boldsymbol{\xi}_n, \boldsymbol{\zeta}_n) = \text{const.} + \underbrace{\ln p(\boldsymbol{y}_n \mid \boldsymbol{\xi}_n) + \ln p(\boldsymbol{z}_n \mid \boldsymbol{\xi}_n)}_{L(\boldsymbol{\xi}_n)} \\ + \sum_{c=1}^{C} \zeta_{nc} \underbrace{\left[\langle \ln v_c \rangle + \frac{1}{2} \sum_{d=1}^{D} \langle \ln \lambda_{cd} \rangle - \left\langle (\xi_{nd} - \mu_{cd})^2 \lambda_{cd} \right\rangle_{q(\mu,\lambda)} \right]}_{l_c(\boldsymbol{\xi}_n)}. \end{aligned} \tag{C.8}$$

The function $L(\boldsymbol{\xi}_n)$ on the first line is the log-likelihood of observed data, given the parameters. The functions $l_c(\boldsymbol{\xi}_n)$ on the second line are the "log-responsibilities", i.e., non-normalized log probabilities that $\boldsymbol{\xi}_n$ was generated by the cth mixture component. This yields a categorical distribution for $\boldsymbol{\zeta}_n$, given $\boldsymbol{\xi}_n$, with normalized probability mass $w_c(\boldsymbol{\xi}_n) = \mathsf{P}(\zeta_{nc} = 1 \mid \boldsymbol{\xi}_n)$. A properly normalized version of this conditional distribution is

$$q(\boldsymbol{\zeta}_n \mid \boldsymbol{\xi}_n) = \prod_{c=1}^{C} w_c(\boldsymbol{\xi}_n)^{\zeta_{nc}}, \quad \text{with } \mathsf{E}(\zeta_{nc} \mid \boldsymbol{\xi}_n) = w_c(\boldsymbol{\xi}_n) = \frac{e^{l_c(\boldsymbol{\xi}_n)}}{\sum_{i=1}^{C} e^{l_i(\boldsymbol{\xi}_n)}} \tag{C.9}$$

The marginal density $q(\boldsymbol{\xi}_n)$ is obtained from (C.8) and (C.9) as

$$\begin{aligned} \ln q(\boldsymbol{\xi}_n) &= \ln q(\boldsymbol{\xi}_n, \boldsymbol{\zeta}_n) - \ln q(\boldsymbol{\zeta}_n \mid \boldsymbol{\xi}_n) = \\ &= \text{const.} + L(\boldsymbol{\xi}_n) + \sum_{c=1}^{C} \zeta_{nc} l_c(\boldsymbol{\xi}_n) - \zeta_{nc} l_c(\boldsymbol{\xi}_n) + \zeta_{nc} \ln \left(\textstyle\sum_{i=1}^{C} e^{l_i(\boldsymbol{\xi}_n)} \right) = \\ &= \text{const.} + L(\boldsymbol{\xi}_n) + \left(\sum_{c=1}^{C} \zeta_{nc} \right) \ln \left(\textstyle\sum_{i=1}^{C} e^{l_i(\boldsymbol{\xi}_n)} \right) = \\ &= \text{const.} + L(\boldsymbol{\xi}_n) + \ln \left(\textstyle\sum_{i=1}^{C} e^{l_i(\boldsymbol{\xi}_n)} \right) \end{aligned} \tag{C.10}$$

Here, the last term is the log likelihood that a candidate sample $\boldsymbol{\xi}_n$ is drawn from *any* of the mixture components in the GMM.

The Hamiltonian sampling procedure needs only the non-normalized part of $\ln q(\boldsymbol{\xi}_n)$ defined in (C.10) and its gradient w.r.t. $\boldsymbol{\xi}_n$, which is straightforward to compute. The sampling must be repeated for each iteration of the variational procedure to account for the current estimate of the population model. The sampling is done separately for each participant model, so the computations for separate partipants can run in parallel processes.

Now, using a set $\{\hat{\boldsymbol{\xi}}_{ns} : s = 1, \ldots, S\}$ of equally probable sample vectors drawn from $q(\boldsymbol{\xi}_n)$, expectations of any function of the variables are consistently approximated simply by an average across all those samples. In particular, the following marginal means will be needed in other parts of the learning procedure:

$$\langle \zeta_{nc} \rangle = \left\langle \langle \zeta_{nc} \mid \boldsymbol{\xi}_n \rangle_{\zeta} \right\rangle_{\boldsymbol{\xi}_n} = \langle w_c(\boldsymbol{\xi}_n) \rangle_{\boldsymbol{\xi}_n} \approx \frac{1}{S} \sum_{s=1}^{S} w_c(\hat{\boldsymbol{\xi}}_{ns}) \tag{C.11}$$

$$\langle \zeta_{nc} \xi_{nd} \rangle \approx \frac{1}{S} \sum_{s=1}^{S} w_c(\hat{\boldsymbol{\xi}}_{ns}) \hat{\xi}_{nsd} \tag{C.12}$$

$$\left\langle \zeta_{nc} \xi_{nd}^2 \right\rangle \approx \frac{1}{S} \sum_{s=1}^{S} w_c(\hat{\boldsymbol{\xi}}_{ns}) \hat{\xi}_{nsd}^2 \tag{C.13}$$

C.2.2 Mixture weights

As (C.1) is a sum of terms involving $\ln v_c$, the variational mixture-weight distribution is defined by

$$\ln q(\boldsymbol{v}) = \text{const.} + \sum_{c=1}^{C} \left(\gamma'_c - 1 + \sum_{n=0}^{N-1} \langle \zeta_{nc} \rangle \right) \ln v_c. \tag{C.14}$$

using the marginal expected mixture weights $\langle \zeta_{nc} \rangle$ from (C.11). Thus, the variational distribution again has the Dirichlet form,

$$q(\boldsymbol{v}) \propto \prod_{c=1}^{C} v_c^{\gamma_c - 1} \tag{C.15}$$

with concentration parameters $\boldsymbol{\gamma} = (\gamma_1, \ldots, \gamma_C)$ updated as

$$\gamma_c = \gamma'_c + \sum_{n=0}^{N-1} \langle \zeta_{nc} \rangle \tag{C.16}$$

C.2.3 Gauss-gamma mixture components

As (C.1) is a sum across terms for every element in every mixture component, the variational distributions are defined by

$$\begin{aligned}
&\ln q(\mu_{cd}, \lambda_{cd}) + \text{const.} = \\
&= \frac{1}{2} \ln \lambda_{cd} - \frac{1}{2} (\mu_{cd} - m'_{cd})^2 \nu' \lambda_{cd} - \frac{1}{2} \sum_{n=0}^{N-1} \left\langle \zeta_{nc} (\xi_{nd} - \mu_{cd})^2 \right\rangle_{\xi,\zeta} \lambda_{cd} \\
&\quad + (a' - 1) \ln \lambda_{cd} - b'_d \lambda_{cd} + \frac{1}{2} \sum_{n=0}^{N-1} \langle \zeta_{nc} \rangle \ln \lambda_{cd} = \\
&= \frac{1}{2} \ln \lambda_{cd} - \frac{1}{2} \underbrace{\left(\nu' + \textstyle\sum_n \langle \zeta_{nc} \rangle \right)}_{\nu_c} \mu_{cd}^2 \lambda_{cd} + \mu_{cd} \underbrace{\left(\nu' m'_{cd} + \textstyle\sum_n \langle \zeta_{nc} \xi_{nd} \rangle \right)}_{\nu_c m_{cd}} \lambda_{cd} \\
&\quad - \frac{1}{2} {m'_{cd}}^2 \nu' \lambda_{cd} - \frac{1}{2} \sum_{n=0}^{N-1} \left\langle \zeta_{nc} \xi_{nd}^2 \right\rangle \lambda_{cd} \\
&\quad + (a' - 1) \ln \lambda_{cd} - b'_d \lambda_{cd} + \frac{1}{2} \sum_{n=0}^{N-1} \langle \zeta_{nc} \rangle \ln \lambda_{cd}
\end{aligned} \tag{C.17}$$

As this is a second-degree polynomial in μ_{cd}, by completing the square we find the logarithm of a conditional Gaussian density for μ_{cd}, given precision λ_{cd}, as

$$\ln q(\mu_{cd} \mid \lambda_{cd}) = \text{const.} + \frac{1}{2} \ln \lambda_{cd} - \frac{1}{2} (\mu_{cd} - m_{cd})^2 \nu_c \lambda_{cd} \tag{C.18}$$

Thus, the variational density for the component mean is again a conditional Gaussian

$$q(\mu_{cd} \mid \lambda_{cd}) = \sqrt{\frac{\nu_c \lambda_{cd}}{2\pi}} e^{-\frac{1}{2}(\mu_{cd} - m_{cd})^2 \nu_c \lambda_{cd}} \tag{C.19}$$

with parameters updated as

$$\nu_c = \nu' + \sum_{n=0}^{N-1} \langle \zeta_{nc} \rangle \tag{C.20}$$

$$m_{cd} = \frac{1}{\nu_c} \left(\nu' m'_{cd} + \sum_{n=0}^{N-1} \langle \zeta_{nc} \xi_{nd} \rangle \right) \tag{C.21}$$

The variational density for the precision parameter is defined by

$$\begin{aligned} \ln q(\lambda_{cd}) = \ln q(\mu_{cd}, \lambda_{cd}) - \ln q(\mu_{cd} \mid \lambda_{cd}) = \\ = \text{const.} + \left(a' - 1 + \frac{1}{2} \sum_{n=0}^{N-1} \langle \zeta_{nc} \rangle \right) \ln \lambda_{cd} \\ - \left(b'_d + \frac{1}{2} {m'_{cd}}^2 \nu' - \frac{1}{2} \nu_c m_{cd}^2 + \frac{1}{2} \sum_{n=0}^{N-1} \left\langle \zeta_{nc} \xi_{nd}^2 \right\rangle \right) \lambda_{cd} \end{aligned} \tag{C.22}$$

This is again the logarithm of a gamma density

$$q(\lambda_{cd}) \propto \lambda_{cd}^{a_c - 1} e^{-b_{cd} \lambda_{cd}} \tag{C.23}$$

with parameters updated as

$$a_c = a' + \frac{1}{2} \sum_{n=0}^{N-1} \langle \zeta_{nc} \rangle \tag{C.24}$$

$$b_{cd} = b'_d + \frac{1}{2} \nu' {m'_{cd}}^2 - \frac{1}{2} \nu_c m_{cd}^2 + \frac{1}{2} \sum_{n=0}^{N-1} \left\langle \zeta_{nc} \xi_{nd}^2 \right\rangle \tag{C.25}$$

C.3 Log-likelihood lower bound

To monitor the progress of variational learning the lower bound (C.3) is most conveniently calculated at each iteration as

$$\begin{aligned} \mathcal{L}(q) = \left\langle \ln \frac{p(\underline{\boldsymbol{y}}, \underline{\boldsymbol{z}}, \underline{\boldsymbol{\xi}}, \underline{\boldsymbol{\mu}}, \underline{\boldsymbol{\lambda}}, \underline{\boldsymbol{\zeta}}, \boldsymbol{v})}{q(\underline{\boldsymbol{\xi}}, \underline{\boldsymbol{\mu}}, \underline{\boldsymbol{\lambda}}, \underline{\boldsymbol{\zeta}}, \boldsymbol{v})} \right\rangle_{q()} = \\ = \sum_n \langle \ln p(\boldsymbol{y}_n, \boldsymbol{z}_n \mid \boldsymbol{\xi}_n) \rangle_\xi + \left\langle \ln p(\boldsymbol{\xi}_n \,\middle|\, \underline{\boldsymbol{\mu}}, \underline{\boldsymbol{\lambda}}, \underline{\boldsymbol{\zeta}}) \right\rangle_q - \langle \ln q(\boldsymbol{\xi}_n) \rangle_q \\ - \left\langle \ln \frac{q(\underline{\boldsymbol{\zeta}} \mid \underline{\boldsymbol{\xi}})}{p(\underline{\boldsymbol{\zeta}} \mid \boldsymbol{v})} \right\rangle_q - \left\langle \ln \frac{q(\underline{\boldsymbol{\mu}}, \underline{\boldsymbol{\lambda}})}{p(\underline{\boldsymbol{\mu}}, \underline{\boldsymbol{\lambda}})} \right\rangle_q - \left\langle \ln \frac{q(\boldsymbol{v})}{p(\boldsymbol{v})} \right\rangle_q \end{aligned} \tag{C.26}$$

This lower bound is theoretically guaranteed to be non-decreasing for each step of the learning procedure, except for minor random variations caused by the sampling approximation. Here the first two terms were already calculated by (C.10) during sampling. The third term is the entropy for each $\boldsymbol{\xi}_n$, which is calculated from the samples using a nearest-neighbour ("Kozachenko-Leonenko") estimator (Singh and Poczos, 2016). The last three terms subtract the Kullback-Leibler divergence KL $(q \,\|\, p)$ between posterior and prior distributions for the four types of population parameters.

The subtraction of Kullback-Leibler divergences represents the cost of model complexity which is the basis of the *Occam's Razor* effect. The variational learning automatically tends to push parameter distributions toward their priors for any mixture component that is not really needed to model the observed data, because this reduces the Kullback-Leibler divergence and increases $\mathcal{L}(q)$. When the learning procedure has finished, any unused mixture components may be deleted from the model with no loss of modeling accuracy.

D Predictive results

The learned population model is used to calculate two marginal distributions:

D.1 Population mean

The marginal distribution of the mean vector $\boldsymbol{\mu} = (\mu_1 \ldots, \mu_D)$ (equal to the median) is a mixture density, based on (C.19) and (C.15), integrated over the variational distributions (C.23) of the precision parameters.

$$p(\boldsymbol{\mu}) = \sum_{c=1}^{C} \langle v_c \rangle \prod_{d=1}^{D} \int q(\mu_{cd} \mid \lambda_{cd}) q(\lambda_{cd}) \, \mathrm{d}\lambda_{cd}$$

$$\propto \sum_{c=1}^{C} \langle v_c \rangle \prod_{d=1}^{D} \left(1 + \frac{(\mu_{cd} - m_{cd})^2 \nu_c}{2 b_{cd}} \right)^{-\frac{2a_c+1}{2}} \quad \text{(D.1)}$$

Thus, the marginal density for the mean is a mixture including a univariate Student-t distribution for each element μ_{cd} of each mixture component, with location m_{cd}, scale $\sqrt{b_{cd}/a_c \nu_c}$, and degrees-of-freedom $2a_c$.

D.2 Random individual

The predictive distribution $p(\boldsymbol{\xi}_N)$ for parameters $\boldsymbol{\xi}_N = (\ldots, \xi_{Nd}, \ldots)$ for a future not-yet-seen individual randomly drawn from the modeled population,

is a mixture density based on (B.2) and (C.15), integrated over the learned variational distributions for the Gaussian mean and precision parameters:

$$p(\boldsymbol{\xi}_N) = \sum_{c=1}^{C} \langle v_c \rangle \prod_{d=1}^{D} \iint \sqrt{\frac{\lambda_{cd}}{2\pi}} e^{-\frac{1}{2}(\xi_{nd}-\mu_{cd})^2 \lambda_{cd}} q(\mu_{cd} \mid \lambda_{cd}) q(\lambda_{cd}) \, \mathrm{d}\mu_{cd} \, \mathrm{d}\lambda_{cd}$$
$$\propto \sum_{c=1}^{C} \langle v_c \rangle \prod_{d=1}^{D} \left(1 + \frac{(\xi_{Nd} - m_{cd})^2 \nu_c}{2 b_{cd}(\nu_c + 1)} \right)^{-\frac{2a_c+1}{2}} \qquad \text{(D.2)}$$

Using the learned variational distributions (C.19) and (C.23) for the Gaussian component mean and precision, the resulting mixture includes a univariate Student-t distribution for each element ξ_{Nd}, given each mixture component with location m_{cd}, scale $\sqrt{b_{cd}(\nu_c + 1)/a_c \nu_c}$, and degrees-of-freedom $2a_c$.

References

Bishop, C. M. (2006). *Pattern recognition and machine learning.* Springer, New York, NY, USA.

Neal, R. M. (2011). MCMC using Hamiltonian dynamics. In Brooks, S., Gelman, A., Jones, G. L., and Meng, X.-L., editors, *Handbook of Markov chain Monte Carlo*, chapter 5, pages 113–162. Chapman and Hall / CRC Press, Boca Raton, FL, USA.

Singh, S. and Poczos, B. (2016). Analysis of k-nearest neighbor distances with application to entropy estimation. *arXiv:1603.08578 [math.ST].*
